# Comparative proteomic analysis reveals a dynamic pollen plasma membrane protein map and the membrane landscape of receptor-like kinases and transporters important for pollen tube growth and interaction with pistils in rice

**DOI:** 10.1186/s12870-016-0961-7

**Published:** 2017-01-05

**Authors:** Ning Yang, Tai Wang

**Affiliations:** Key Laboratory of Plant Molecular Physiology, Institute of Botany, Chinese Academy of Sciences, and National Center for Plant Gene Research, 20 Nanxincun, Xiangshan, Haidianqu, Beijing, 100093 China

**Keywords:** Plasma membrane, Receptor-like kinase, Transporters, Quantitative proteomics, iTRAQ, Pollen-pistil interaction, Rice

## Abstract

**Background:**

The coordination of pollen tube (PT) growth, guidance and timely growth arrest and rupture mediated by PT-pistil interaction is crucial for the PT to transport sperm cells into ovules for double fertilization. The plasma membrane (PM) represents an important interface for cell–cell interaction, and PM proteins of PTs are pioneers for mediating PT integrity and interaction with pistils. Thus, understanding the mechanisms underlying these events is important for proteomics.

**Results:**

Using the efficient aqueous polymer two-phase system and alkali buffer treatment, we prepared high-purity PM from mature and germinated pollen of rice. We used iTRAQ quantitative proteomic methods and identified 1,121 PM-related proteins (PMrPs) (matched to 899 loci); 192 showed differential expression in the two pollen cell types, 119 increased and 73 decreased in abundance during germination. The PMrP and differentially expressed PMrP sets all showed a functional skew toward signal transduction, transporters, wall remodeling/metabolism and membrane trafficking. Their genomic loci had strong chromosome bias. We found 37 receptor-like kinases (RLKs) from 8 kinase subfamilies and 209 transporters involved in flux of diversified ions and metabolites. In combination with the rice pollen transcriptome data, we revealed that in general, the protein expression of these PMrPs disagreed with their mRNA expression, with inconsistent mRNA expression for 74% of differentially expressed PMrPs.

**Conclusions:**

This study identified genome-wide pollen PMrPs, and provided insights into the membrane profile of receptor-like kinases and transporters important for pollen tube growth and interaction with pistils. These pollen PMrPs and their mRNAs showed discordant expression. This work provides resource and knowledge to further dissect mechanisms by which pollen or the PT controls PMrP abundance and monitors interactions and ion and metabolite exchanges with female cells in rice.

**Electronic supplementary material:**

The online version of this article (doi:10.1186/s12870-016-0961-7) contains supplementary material, which is available to authorized users.

## Background

Pollen, consisting of the large vegetative cell (VC) and immobile male gametes (sperm cells) enclosed in the VC, represents an innovative phenotype of plants during evolution. Pollen is tolerant to dehydration and can transport the male gamete over a long distance with the help of wind and/or animals, important forces driving the distribution of plants in the land. Once landing on the stigma of pistils, pollen hydrates, germinates and gives rise to a tip-growing pollen tube (PT). The tube further journeys within the pistil and finally arrives in the synergids of embryo sacs, where it arrests growth and ruptures to release sperm cells for double fertilization. This process requires maintenance of PT integrity and PT–pistil interaction to guide the PT toward the embryo sac and regulate the timely growth arrest of the PT and rupture for immobile gamete release, thereby precisely guaranteeing the one PT to one ovule relationship [1].

The plasma membrane (PM) represents the semi-permeable barrier for selective flux of ion and metabolites across the membrane and an important interface for cell–cell interaction. PM proteins, especially receptor-like kinases (RLKs) and transporters/channels of PTs are the pioneers for mediating PT integrity and interaction with pistils [1]. In *Arabidopsis*, ANX1 and ANX2, members of the plant-specific RLKs of the *Catharanthus roseus* RLK1-like (CrRLK1L) subfamily [2], are localized in the PT PM and redundantly regulate PT growth and integrity [3, 4]. PTs in *anx1anx2* double mutants show precocious rupture. Overexpression of *ANXs* caused PT growth inhibition [5]. RUPO, the rice CrRLK1L member, controls PT growth and integrity by interacting with K^+^ transporters, so a novel RLK signaling pathway mediates K^+^ homeostasis is required for PT growth and integrity [6]. Studies have also revealed the involvement of the leucine-rich repeat RLK (LRR-RLK) subfamily in PT growth and guidance. Tomato pollen-specific LRR-RLKs LePRK1 and LePRK2, expressed specifically in pollen, regulate PT growth and can bind STIGMA-SPECIFIC PROTEIN1 (STIG1), a small cysteine-rich protein from the pistil [7]. The *Arabidopsis* LRR-RLKs MDIS1, MIK1 and MIK2 form heteromers, and the complex functions as a receptor of LURE1, the defensin-like cysteine-rich peptide from synergids, to regulate PT guidance and perception [8]. Another *Arabidopsis* LRR-RLK, PRK6, was identified as a LURE1 receptor and functions in guiding PT tip growth [9]. Furthermore, several other female factors were identified to be involved in PT growth and/or guidance. Tobacco transmitting tissue-specific (TTS), a pistil transmitting tissue-specific arabinogalactan protein, may have roles in guiding PT growth [10]. Chemocyanin, a stigma-expressed small cell wall protein in lily, induces PT chemotropism [11]. ZmEA1, the small protein exclusively expressed in maize egg apparatus, helps guide PT growth in the short range [12]. Thus, additional RLKs are involved in sensing these female factors.

Ion fluxes across the PM and the ion gradient in PTs are well known and are crucial for PT growth [13]. In *Arabidopsis*, AtACA9, a Ca^2+^ATPase for Ca^2+^ efflux is required for PT growth and PT–synergid contact [14]. *Arabidopsis* cyclic nucleotide gated Ca^2+^ channel 7 (AtCNGC7) and 8 for Ca^2+^ influx redundantly regulate the initiation of PT tip growth [15]. AtCNGC18 is essential for PT directional growth *in vitro* [16]. Disruption of SPIK, an inward K^+^ channel in *Arabidopsis*, strongly reduced K^+^ influx, which resulted in impaired pollen germination and PT growth [17]. PTs lacking the cation/proton exchangers CHX21 and CHX23 grew down in the transmitting tract and failed to turn to the ovule [18]. The maize ZmES4, the synergid-expressed defensin-like cysteine-rich protein, has roles in opening the PT PM-localized K^+^ influx channel KZM1, which led to excessive influx of K^+^, thereby causing PT rupture [19]. Moreover, the rice receptor-like kinase RUPO–K^+^ transporter signaling pathway has been revealed in PTs [6].

Despite these promising findings, our knowledge of RLKs and transporters/channels (hereafter called transporters) that function in PT growth and interaction with pistils is limited, and a detailed understanding of these components at the omic-wide level and proteomic characteristics of pollen and PT PM is lacking. A systematic knowledge of RLKs and transporters in the PM is crucial for an in-depth understanding of the mechanisms underlying PT growth and interaction with pistils.

Here, we prepared PMs from mature pollen grains (MPGs) and germinated pollen grains (GPGs) and dissected PM proteins by using iTRAQ quantitative proteomics. We identified 1,121 PM-related proteins (PMrPs) (matched to 899 loci), with 192 differentially expressed during pollen germination, and revealed 37 RLKs and 209 transporters in the proteome. All PMrPs and differentially expressed PMrPs featured signal transduction, transporters, wall remodeling/metabolism and membrane trafficking functions. Further comparison of proteomic and transcriptomic data revealed that PMrPs are in general discordant with their mRNA levels, with inconsistent mRNA profiles for 74% of differentially expressed PMrPs. These results provide insights into the proteomic characteristics of pollen PM and the profile of RLKs and transporters in the membrane.

## Methods

### Pollen collection and in vitro germination

Rice cultivar Zhonghua 10 (*Oryza sativa* L. ssp. *japonica*) was planted under natural conditions in Beijing. Mature pollen grains (MPGs) were collected at anthesis stage by using a modified vacuum cleaner outfitted with nylon meshes. For germination experiments, fresh collected MPGs were transferred into liquid germination medium (40 mg/L H_3_BO_3_, 3 mM Ca (NO_3_)_2_ · 4H_2_O, 3 mg/L VB_1_, 10% PEG4000, 250 mM sucrose) immediately and cultured with gentle shaking at room temperature (~30 °C) for about 15 min. Under this condition, more than 90% of MPGs synchronously germinated to generate polar-growing PTs. Germinated pollen grains (GPGs) were collected by centrifugation at 1000 × g at 4 °C for 5 min. All collected MPGs and GPGs were used immediately or stored at −80 °C.

### Plasma membrane preparation

MPGs and GPGs were homogenized in extracting buffer (250 mM sucrose, protease inhibitor cocktail, 1 mM EDTA, 1 mM DTT, 1 mM PMSF, and 50 mM MOPS/KOH, pH 7.8) by use of the high-speed bench top homogenizer FastPrep-24 (MP Biomedicals, USA). The homogenate was differentially centrifuged at 1,500 × g for 5 min, 12,000 × g for 20 min and then 31,000 × g for 15 min to remove cell debris, mitochondria and other organelle contaminants, respectively. The resulting supernatant was centrifuged at 100,000 × g for 1 h with use of BECKMAN Optima L-80XP (70Ti Rotor, Beckman Coulter, USA) to collect pellets (total microsomal vesicles [MSVs]). MSVs were resuspended in PM isolation buffer (250 mM sucrose, 1 mM DTT, 1 mM PMSF, and 5 mM potassium phosphate, pH 7.8) and used to enrich PM vesicles by use of an aqueous polymer two-phase system [20] of 6.5% (w/w) PEG3350 (Sigma), 6.5% (w/w) Dextran T-500 (Pharmacia), 250 mM sucrose, 5 mM KCl, 1 mM DTT, and 5 mM potassium phosphate, pH 7.8. After enrichment, the collected upper phase was diluted more than three-fold with dilution buffer (250 mM sucrose, 1 mM DTT, 1 mM PMSF, and 50 mM MOPS/KOH, pH 7.8) and centrifuged at 200,000 × g for 1 h to collect PM vesicles. PM vesicles were washed with the dilution buffer, then treated with 100 mM sodium carbonate (pH 11.5) to remove soluble proteins associated with the PM vesicles as described [21]. All procedures were carried out at 4 °C. Protein concentration was measured by Bradford assay with bovine serum albumin (BSA) as a standard.

### SDS-PAGE and western blot analysis

Proteins were separated by 10% SDS-PAGE. For Western blot analysis, proteins in gels were electrotransferred onto a PVDF membrane (Pierce, USA) with 25 mM Tris, 192 mM glycine and 20% methanol and incubated with the primary rabbit antibodies for PM H^+^-ATPase (PMA2) from *Nicotiana plumbaginifolia* (1:5000 dilution) [22]; mitochondrial cytochrome oxidase subunit 2 (COX II) (Agrisera no. AS04053A, Sweden, 1:5000 dilution), vacuole ATPase (V-ATPase) (Agrisera no. AS07213, 1:5000 dilution), 40S ribosomal protein S14-1 (Agrisera no. AS09477, 1:3000 dilution), and ras-related protein1 (Sar) (Agrisera no. AS08326, 1:1000 dilution) from *Arabidopsis thaliana*; histone H1 (LOC_O s04g18090.1, Beijing Protein Innovation, China, 1:1000 dilution), DEAD-box ATP-dependent RNA helicase (eIF4a; LOC_Os02g05330.1, 1:1000 dilution), glyceraldehyde-3-phosphate dehydrogenase (GAPDH; LOC_Os04g40950.1, 1:1000 dilution), and flotillin like protein (Band_7; Beijing B&M Biotech Co., 1:1000 dilution) from *Oryza sativa*. Optical density of Western blot bands was quantified by using Image-Pro Plus v6.0 (Media Cybernetics, USA).

### In-solution digestion, iTRAQ labeling and strong cation exchange fractionation

Protein digestion and iTRAQ labeling were performed according to the iTRAQ reagents chemistry reference guide (iTRAQ Reagents Multiplex kit, AB SCIEX) with a few modifications. Briefly, proteins (100 μg) from purified PM vesicles were supplemented with *Rapi*Gest SF surfactant (Waters, USA) at a final concentration of 0.2% (w/v) for denaturation and enzymatic digestion enhancing, then reduced with 10 mM TCEP, pH 8.0 at 56 °C for 1 h followed by alkylation with 50 mM iodoacetamide in the dark (room temperature, 45 min). Pretreated proteins were digested with trypsin at a ratio of 1:50 (w/w) (Roche) at 37 °C for 16 h, and resulting peptides were labeled with iTRAQ reagents. Experimental repeats were designed as follows: experiment 1 was a mixture of 115 tag-labeled MPG and 117 tag-labeled GPG samples; experiment 2 was a mixture of 117 tag-labeled MPG and 115 tag-labeled GPG samples. Experiment 1 and 2 were lyophilized and subjected to strong cation exchange (SCX) fractionation.

SCX fractionation was performed with an AKTA Purifier 10 HPLC system (GE Amersham Biosciences, USA). The lyophilized samples were resuspended in solvent A (5 mM ammonium chloride, 25% [v/v] acetonitrile, pH 3.0) and fractionated with a PolySULFOETHYL A column (2.1 × 200 mm, 5 μm, 300 Å, PolyLC, Columbia, MD, USA) at a flow rate of 200 μl/min through a linear gradient (0-60%, 90 min) of solvent B (500 mM ammonium chloride, 25% [v/v] acetonitrile, pH 3.0) followed by 60-100% solvent B for 10 min, and 100% solvent B for 15 min. Each separated sample was pooled to 16 fractions and lyophilized for LC-MS/MS analysis.

### Nano LC-MS/MS analysis

Each SCX faction was reconstituted with 100 μL 0.1% formic acid in water. After the removal of salt on a Vydac C18 SPE cartridge by centrifugation, the desalted fraction was supplemented with 50 μL 60% acetonitrile and dried with a speed-vac. Each dried fraction was solved in 20 μL 0.1% formic acid, and 10 μL of the solution was injected for nanoLC-MS/MS analysis by use of AB SCIEX Triple TOF 5600 MS (Concord, Ontario, Canada) equipped with a splitless Eksigent nano Ultra 2D Plus nanoLC system and a cHiPLC-Nanoflex microchip system (Dublin, CA, USA). The cHiPLC-system used changeable microfluidic traps (200 μm × 5 mm) and analytical columns (75 μm × 150 mm) packed with ChromXP C18 (3 μm, 120 Å) for online separation analysis. Sample loading, trapping and desalting involved 100% of mobile phase A (2% acetonitrile, 0.2% formic acid, 98% water) at a flow rate of 2 μL/min for 10 min. Peptide elution was started with 5% mobile phase B (98% acetonitrile, 0.2% formic acid, 2% water), then the gradient increased linearly to 24% in 70 min at a flow rate of 300 nL/min. The total gradient length was 120 min. MS data acquisition was performed in the information dependent acquisition (IDA) mode. Triple TOF 5600 MS was operated with a resolving power of 30,000 (FWHM) for TOF MS scans. IDA survey scans were acquired in 250 ms with mass range of m/z 350–1250. As many as 30 product ion scans were collected for 100 ms with mass range of m/z 100–1500, if exceeding a threshold of 120 cps (counts/s) and with a charge state of +2 to +5. Dynamic exclusion was set for 18 sec. Collision energies were calculated on-the-fly for all precursor ions by using empirical equations based on mass and charge (Rolling CE on), and the Enhance iTRAQ function was turned on to improve the efficiency of the collision-induced dissociation.

### Protein identification and quantitation

The raw data files (*.wiff) generated by Triple TOF 5600 were analyzed by using ProteinPilot 4.0 (revision 460, AB SCIEX), which involved two different algorithms; Paragon and Pro Group.

Paragon is a search engine that uses feature probabilities and sequence temperature values to identify peptides from MS/MS spectra [23]. Database and parameters used for searching were as follows: NCBI *Oryza sativa* nonredundant database (136,389 protein entries, August 2011); Sample Type-iTRAQ 4plex labeled; Cys Alkylation-Iodoacetamide; Digestion-Trypsin; Instrument-Triple TOF 5600; Quantitate; Bias Correction; Background Correction; Biological modifications. Precursor mass tolerance was 0.05 Da and fragment mass tolerance was 0.1 Da. As part of the Paragon analysis method, false discovery rate analysis was performed by searching the decoy database to assess the rate of inaccurately identified proteins.

Paragon search results were further processed by the Pro Group algorithm to determine the smallest justifiable set of detected proteins. Each detected protein has an unused protein score, a measurement of all the peptide evidence for a protein that is not better explained by a higher ranking protein, and this score is the true indicator of protein confidence. Unused protein scores 2.0, 1.3, 1.0, and 0.47 correspond to peptide confidence 99, 95, 90, and 66% respectively (score 1.3, 95% confidence was threshold of this work), as shown in the ProteinPilot Software Beta Help (AB SCIEX).

Protein quantitative analysis was also performed by use of ProteinPilot 4.0. The software calculates protein expression change ratios between different samples based on the relative intensities of iTRAQ-labeled peptides. Only ratios from the spectra that are distinct to each protein or protein form were used, to eliminate any masking of changes in expression due to peptides shared between proteins. For each protein expression change ratio reported, the program calculates a p-value that indicates the probability of randomly detecting a ratio different from 1. If an expression change ratio is extremely well determined, a real change can be detected even when the ratio is not very different from 1. To obtain a more accurate quantified result, we chose bias correction and background correction when searching the database by Paragon. Specifically, the criteria for determining MPGs and GPGs differentially expressed PM proteins are two experiments expressed, *p*-value ≤ 0.05 and GPG/MPG ≥ 1.50 or GPG/MPG ≤ 0.67.

### *In silico* analysis

Protein molecular weight (MW) and isoelectric point (pI) were calculated by using the ProParam tool of Expasy (http://web.expasy.org/protparam/). The matched loci IDs were obtained from the Rice Genome Annotation Project (http://rice.plantbiology.msu.edu/index.shtml). Cellular component, biological process, and molecular function for proteins were annotated by using gene ontology (GO, http://www.geneontology.org/) or WoLF PSORT (http://www.genscript.com/psort/wolf_psort.html). Protein transmembrane domains (TMDs) were predicted by using HMMTOP 2.0 (http://www.enzim.hu/hmmtop/). Modifications related to membrane localization of a protein including glycosylphosphatidyl inositol (GPI) attachment, prenylation, myristoylation, and palmitoylation were predicted by using the big-PI Predictor (http://mendel.imp.ac.at/gpi/gpi_server.html), PrePS-Prenylation Prediction Suite (http://mendel.imp.ac.at/PrePS/), N-Myristoyltransferase (NMT, http://mendel.imp.ac.at/myristate/), and CSS-Palm 3.0 (http://csspalm.biocuckoo.org/), respectively. Protein annotations were comprehensively evaluated by using a combination of NCBI (http://www.ncbi.nlm.nih.gov/), RGAP 7 (http://rice.plantbiology.msu.edu/), and ARAMEMNON 7.0 (http://aramemnon.uni-koeln.de/).

### Sequence and phylogenetic analysis

Protein sequences of *Arabidopsis* were obtained from The *Arabidopsis* Information Resource and those for rice and other species were obtained from NCBI. Multiple sequence alignments involved use of BioEdit with the Clustal W method. The protein relevance and phylogenetic tree analysis involved use of MEGA4.0 software.

## Results

### Preparation and purity detection of plasma membranes from pollen

**Fig. 1:**
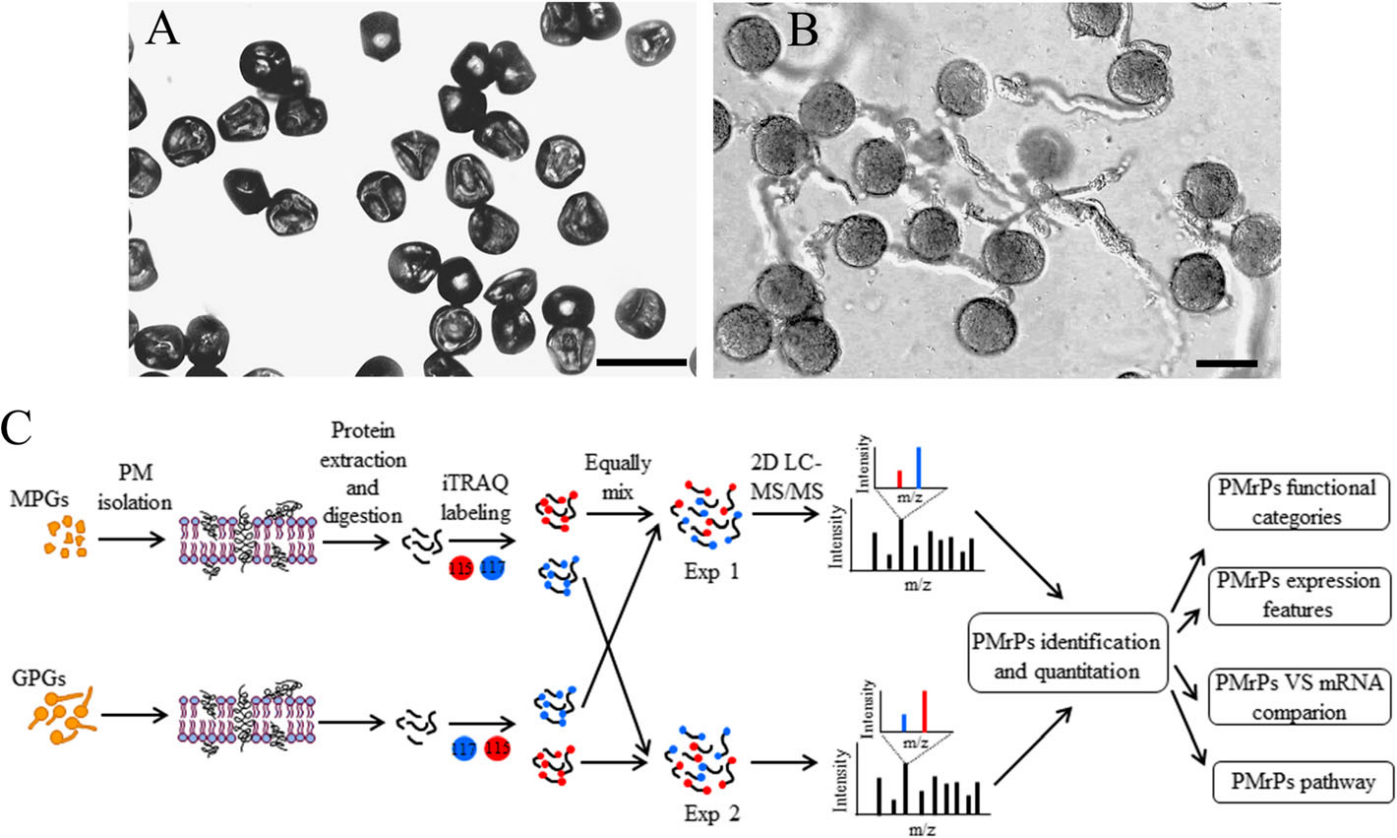
Morphology of rice mature pollen grains (MPGs) and germinated pollen grains (GPGs) and the overview of workflow. **a** Highly dehydrated MPGs. **b** *in vitro* germinated GPGs. **c** Overview of the experimental scheme. Bar = 50 μm

**Fig. 2:**
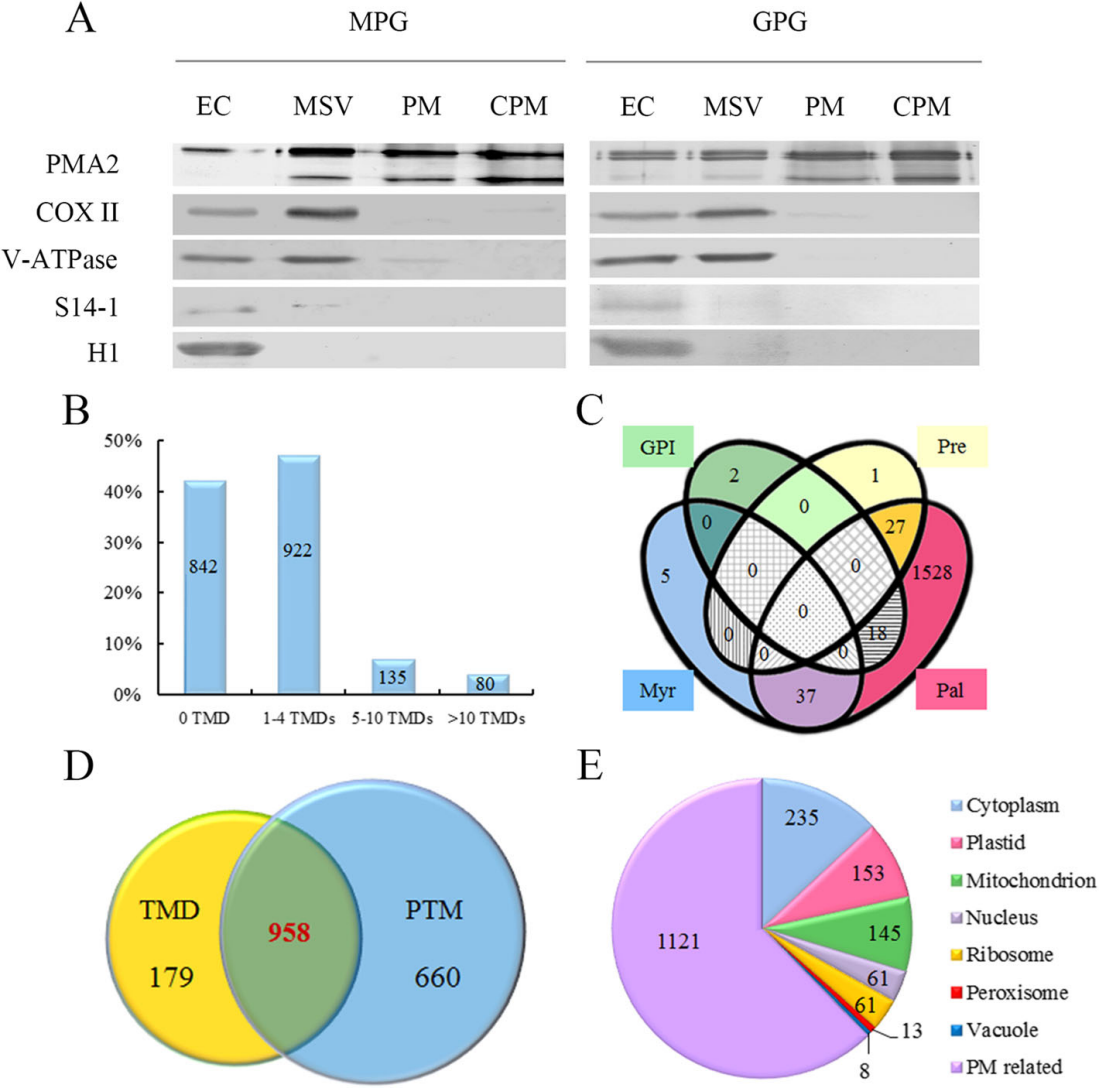
Plasma membrane (PM) purity verification and PM-related proteins evaluation. **a** Western blot examination of plasma membrane enrichment. Proteins from entire cell (EC) lysates, microsomal vesicles (MSV), PM (plasma membrane vesicles) and carbonate-washed PM (CPM) were separated by 10% SDS-PAGE, transferred to PVDF membranes and detected with antibodies for PMA2, a PM marker; COXII, a mitochondrial marker; V-ATPase, a vacuole marker; S14-1, a ribosome marker; or H1, a nucleus marker. For detection of PMA2, COXII and V-ATPase, 5 μg protein was loaded per lane; for detection of S14-1 and H1, 10 μg protein was loaded per lane. **b** Summary of proteins with transmembrane domain (TMD) predicted with use of HMMTOP 2.0. **c** Venn diagram depicting the distribution of proteins with different lipid modifications. GPI, glycosylphosphatidylinositol anchor; Pre, prenylation site; Myr, myristoylation site; Pal, palmitoylation site. **d** Proteins predicted to have a TMD or post-translational modification (PTM) or both. **e** Protein subcellular locations annotated by Gene Ontology or WoLF PSORT showed that 1,121 of the 1,797 proteins with a TMD and/or PTM had PM location information

To identify PM proteins, we first prepared PM vesicles from rice MPGs (Fig. 1a) and GPGs (Fig. 1b) by using the aqueous two-phase partition system followed by a high pH carbonate buffer wash (Fig. 1c). Furthermore, we evaluated the purity of the purified PMs by Western blot analysis with antibodies for PM-specific P-type H^+^-ATPase PMA2, mitochondrial COX II, vacuole-specific V-ATPase, ribosome protein S14-1, and nuclear protein histone H1. PMA2 was detected as two different isoforms in rice pollen: one showed increased abundance from the entire cell (EC) lysate to carbonate buffer-washed PM (CPM) and one was almost undetectable in EC lysates but was most abundant in CPM (Fig. 2a). All other marker proteins were abundant in EC lysates and/or microsome vesicles and almost undetectable in CPM. These results indicated high purity of the purified PMs with high pH carbonate buffer treatment.

### Protein identification and PM-related protein evaluation

To identify PM-related proteins and determine differences in PM proteomes between MPGs and GPGs, we digested PM proteins with trypsin by using *Rapi*Gest SF and obtained well-digested peptides (Additional file 1) for iTRAQ labeling. iTRAQ-labeled peptides were fractioned into 16 fractions by SCX chromatography to reduce sample complexity and increase the identification efficiency of low-abundant PM proteins. The UV-Time curves showed high reproducibility between biological repeated experiments (Additional file 2).

Proteins in these fractions were analyzed by reverse-phase high-performance liquid chromatography coupled with MS/MS. Under the criterion of false discovery rate (FDR) < 1%, unused ≥ 1.3 and two or more unique peptides matched, we identified 1,474 proteins with FDR 0.07% in experiment 1, and 1,284 proteins with FDR 0.08% in experiment 2 (Additional file 3). In total, 1,979 proteins were identified (matched to 1,631 loci), of which 779 were shared in both experiments, with 695 only in experiment 1 and 505 in experiment 2 (Additional file 4).

We analyzed PM location information for the identified 1,979 protein according to annotation of transmembrane domain (TMD), posttranslational modification (PTM), and subcellular location. The analysis of TMDs showed that 1,137/1,979 proteins (57.5%) had at least one TMD (1–4 TMDs for 922 proteins, 5–10 for 135, 10–20 for 80) (Fig. 2b). PTMs including GPI-anchor, prenylation, myristoylation, and palmitoylation are important in mediating PM localization of proteins and in regulating stability and function of proteins [24]. Among the 1,979 proteins, 20 (1.0%) had potential GPI-anchor motifs, 28 (1.4%) potential prenylationsites, 42 (2.1%) potential myristoylation sites and 1,610 (81.4%) potential palmitoylation sites (Fig. 2c and Additional file 4). Studies have revealed that palmitoylation plays a key role in protein sorting [25]. Ultimately, our analysis revealed 1,618/1,979 proteins (82%) with membrane-anchoring motifs (Fig. 2c). In total, 1,797/1,979 proteins (91%) were predicted to have TMDs or one or more membrane-anchoring motifs, or both, which represented potential PM proteins in pollen (Fig. 2d). Furthermore, we obtained protein subcellular location information annotated by gene ontology or WoRF PSORT; among the 1,797 proteins, 1,121 showed PM localization, with the remaining 676 having information for localization in cytoplasm, plastid, mitochondria, nuclei, ribosome, peroxisome or vacuole (Fig. 2e and Additional file 4). Therefore, we considered the 676 proteins as possible contaminants, although they or some also possibly localized in the PM. We finally revealed 1,121 proteins (matched to 899 loci) showing a strong relationship with the PM (Fig. 2e and Additional file 5) and used them for the following analysis.

### Functional categories of PM-related proteins

**Fig. 3:**
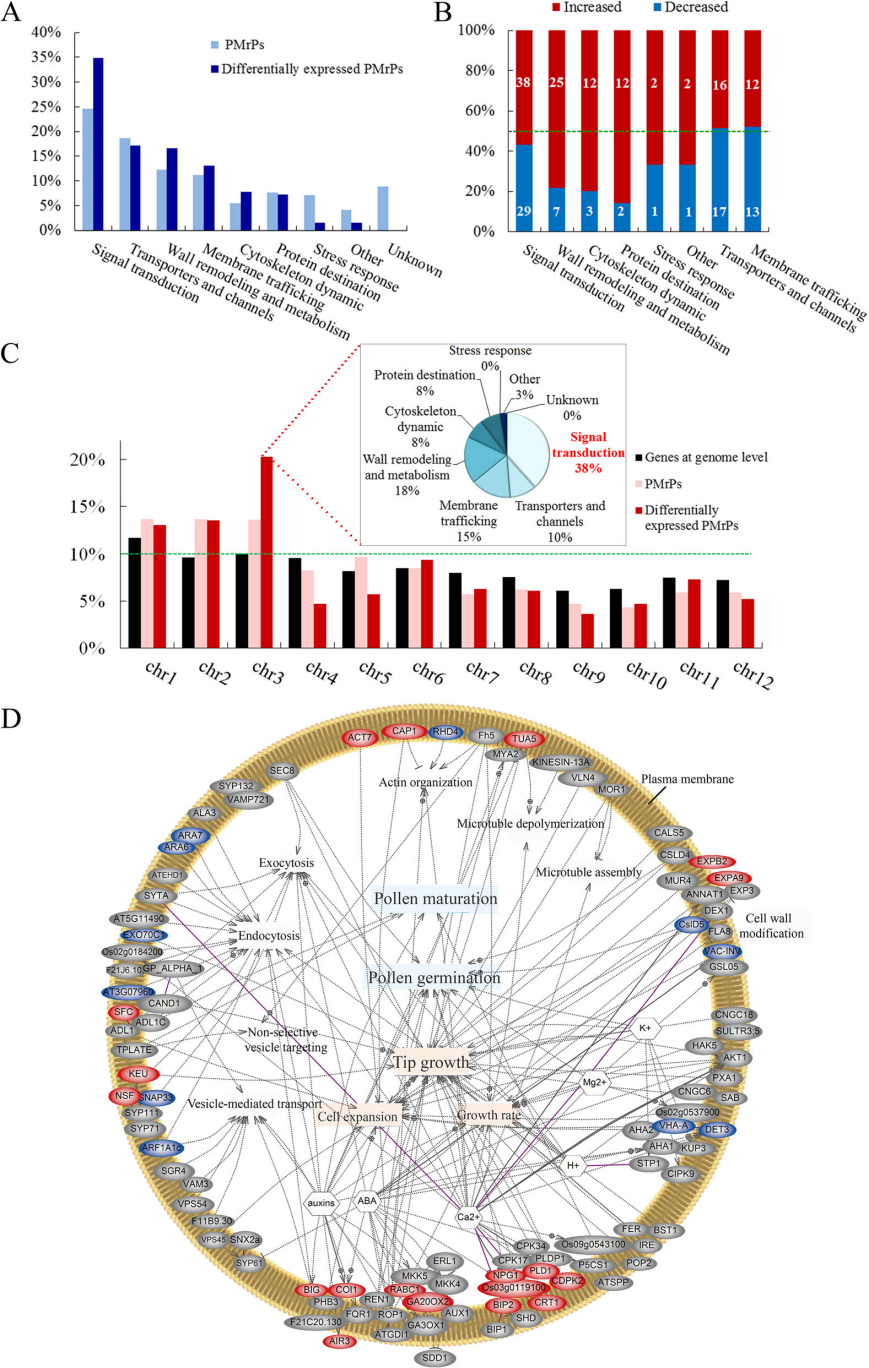
Functional categories and network of PM-related proteins (PMrPs) and differentially expressed PMrPs. **a** Proportion of 1,121 PMrPs and 192 differentially expressed PMrPs in each functional category. **b** Number of abundance-increased and -decreased PMrPs in germinated pollen in each functional category. **c** PMrPs and differentially expressed PMrPs were preferentially encoded by chromosomes (chr) 1, 2 and 3. The proteins biased in chr 3 were significantly skewed toward signal transduction as well as wall remodeling and metabolism, and membrane trafficking. **d** a network of PMrPs. Proteins in red are increased and in blue are decreased in levels in GPGs. Detailed information for the proteins’ abbreviation were listed in Additional file 5A

We collected function information for the 1,121 PM-related proteins (PMrPs) from NCBI, RGAP, ARAMEMNON, and GO databases and analyzed by functional categories to gain insight into the biological process occurring in or around pollen PM. These proteins could be organized into 9 categories (Additional file 5). Overall, 67% of these proteins were in 4 groups: signal transduction (25%), transporters (19%), membrane trafficking (11%) and wall remodeling and metabolism (12%); 24% were associated with 4 other groups (cytoskeleton dynamic, protein destination, stress response, and other process); and the function of the remaining 9% was unknown (Fig. 3a). The preferred distribution of these PMrPs in signal transduction, transporters, membrane trafficking and wall dynamics and metabolism is consistent with important roles of these processes in PT growth and cell–cell interaction during fertilization.

### Quantitative difference between MPG and GPG PMrPs

Our iTRAQ analysis showed high protein quantitative efficiency. Overall, 1,381/1,474 proteins (94%) identified in experiment 1 were quantified and 1,147/1,284 (89%) in experiment 2 were quantified (Additional file 3). Experiments 1 and 2 shared 728 proteins with quantified information. The Pearson correlation coefficient for the two independent iTRAQ experiments was 0.877 (Additional file 6), which indicates well-quantified reproducibility (Additional file 7).

Of the 1,121 PMrPs, 446 (matched to 442 loci) were reproducibly identified in the two independent experiments. Using the cut-off of fold change in expression (GPG/MPG) ≥ 1.5 or ≤0.67 and *p*-value ≤0.05, we revealed 192 PMrPs with significantly changed expression (matched to 192 loci) between MPGs and GPGs, with 119 abundance-increased and 73 abundance-decreased in GPGs (Additional file 8). Among these changed proteins, proteins involved in signal transduction were overrepresented (35%), with a high proportion of proteins related to transporters (17%), wall remodeling and metabolism (17%) and membrane trafficking (13%); the remaining were implicated in cytoskeleton dynamics (8%), protein destination (7%), stress response (2%) and other processes (2%) (Fig. 3a). Most of the proteins implicated in signal transduction (38/67), wall remodeling and metabolism (25/32), cytoskeleton dynamic (12/15), and protein destination (12/14) showed increased expression in GPGs; the number of abundance-increased and -decreased proteins in transporters (16 vs 17) and membrane trafficking (12 vs 13) seemed similar (Fig. 3b).

The rice genome is estimated to encode 50,000 to 60,000 genes. Annotations from RGAP 7.0 (http://rice.plantbiology.msu.edu/annotation_pseudo_current.shtml) showed 12% of these genes distributed in chromosome (chr) 1; 10% each in chr 2, 3, and 4; 8% each in chr 5, 6, 7 and 8; 6% each in chr 9 and 10, and 7% each in chr 11 and 12. The genomic loci of pollen PMrPs and the differentially expressed PMrPs showed a significant chromosome bias. They were enriched on chr 1, 2 and 3, but not on the other chromosomes (Fig. 3c). These enriched proteins on the 3 chromosomes were significantly represented by signal transduction proteins (Fig. 3c, Additional file 9).

**Fig. 4:**
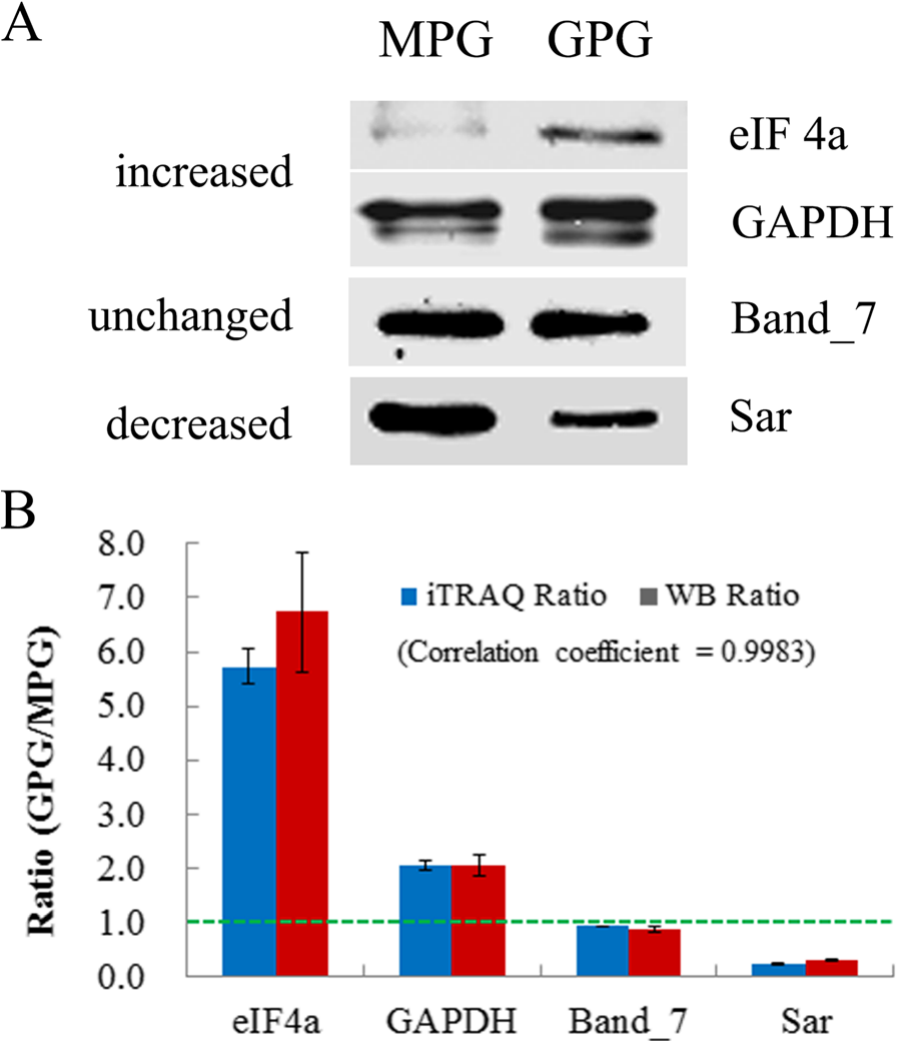
Western blot evaluation of the iTRAQ quantitative information. **a** Western blot analysis of the expression patterns of eIF4a (the eukaryotic initiation factor-4a, gi|115444197), GAPDH (glyceraldehyde-3-phosphate dehydrogenase, gi|115459078), Band_7 (flotillin like protein, gi|48716660) and Sar (ras-related protein, gi|115436368) in MPGs and GPGs. **b** Proteins examined by Western blot analysis and iTRAQ show similar tendency in expression pattern

To validate the expression patterns of proteins detected by the iTRAQ proteomic approach, we used Western blot analysis to examine the expression of 4 proteins that were increased (eIF4a and GAPDH) and decreased (Sar) in levels in GPGs or had no change in level (Band_7) between MPGs and GPGs in iTRAQ data (Fig. 4a). Signal intensity values of Western blot bands were used for quantity analysis (Additional file 10). The expression patterns for all 4 proteins were consistent with the detection by iTRAQ analysis, with a correlation coefficient of 0.9983 (Fig. 4b), thus indicating the reliability of the iTRAQ proteomic results.

### RLKs in pollen plasma membrane

Our analysis revealed 277 PM-related components of signal transduction. These proteins are implicated in diverse signaling pathways, such as Ca^2+^, phospholipid, auxin, abscisic acid (ABA), gibberellic acid (GA) and phosphorylation cascades (Fig. 3d, Additional file 5). RLKs are important regulators of diverse cellular and developmental processes, such as pollen-stigma recognition [26], cell elongation [27], PT guidance [28] and rupture [4]. With a blast-based search of these predicted rice RLK sequences [29], we found 916 unique RLKs annotated in RGAP 7.0; 99 RLKs were in the reported MPG/GPG transcriptome and could be assigned to 11 subfamilies and 1 unassigned group (Additional file 11). Furthermore, 37 RLKs in our pollen PMrP dataset were assigned to the subfamilies CrRLK1L (*n* = 3), extensin (*n* = 1), leucine-rich repeat (LRR-RLK, *n* = 15), proline-rich extensin like receptor kinase (PERK, *n* = 2), receptor-like cytoplasmic kinases (RLCK, *n* = 13), S-domain (SD-RLK, *n* = 1) and unknown receptor kinase (URK, *n* = 1) with 1 not assigned (Table 1). These RLKs had diverse functions in different cellular processes (Additional file 11). Among the 37 pollen PM-localized RLKs, one CrRLK1L and 2 RLCKs were increased, and 2 LRR-RLKs and one RLCK were decreased in abundance in GPGs (Table 1).

**Table 1:** Thirty seven receptor-like kinases (RLKs) identified in plasma membrane of mature pollen grains/germinated pollen grains (MPGs/GPGs) in rice

RLK subfamily	Protein no.	Accession	Matched Locus	MW (Da)	PI	TMD	Change in abundance (GPG/MPG)
CrRLK1L	1	gi|56783691	LOC_Os05g20150	96285.50	5.70	1	–
	2	gi|115462979	LOC_Os05g20150	94782.20	5.87	2	–
	3	gi|25553554	LOC_Os06g03610	90999.30	5.49	3	2.07
Extensin	4	gi|218187906	LOC_Os01g14932	173555.60	6.15	3	–
LRR	5	gi|115464509	LOC_Os05g40200	69908.70	8.81	1	–
	6	gi|13324792	LOC_Os03g50450	71888.10	8.79	2	–
	7	gi|7573610	LOC_Os01g12390	68306.90	8.62	1	–
	8	gi|15128407	LOC_Os01g60330	69220.70	8.66	1	–
	9	gi|125547150	LOC_Os11g26130	98211.90	5.75	3	–
	10	gi|115477354	LOC_Os08g40990	75836.40	6.25	4	–
	11	gi|222640883	LOC_Os08g40990	68833.10	9.62	4	–
	12	gi|49388978	LOC_Os02g07810	72525.70	9.09	2	0.42
	13	gi|52075918	LOC_Os06g45240	72801.70	9.36	2	0.41
	14	gi|115480655	LOC_Os09g38700	74411.20	5.91	1	–
	15	gi|115486303	LOC_Os11g40550	72362.10	6.77	3	–
	16	gi|4680345	LOC_Os11g40550	70125.50	7.72	3	–
	17	gi|125531685	LOC_Os10g22860	116401.40	7.96	1	–
	18	gi|125561357	LOC_Os08g28870	103328.50	5.74	1	–
	19	gi|218201938	LOC_Os09g15700	88665.30	6.47	1	–
PERK	20	gi|24421681	LOC_Os03g12570	194078.50	5.61	4	–
	21	gi|53982302	LOC_Os05g12680	51589.60	6.41	1	–
RLCK	22	gi|115437912	LOC_Os01g39970	84033.50	8.11	0	–
	23	gi|115444273	LOC_Os02g05820	86192.80	6.21	0	–
	24	gi|115445049	LOC_Os02g12660	81207.50	7.52	0	–
	25	gi|115449121	LOC_Os02g54590	84753.10	6.96	1	–
	26	gi|125583945	LOC_Os02g54590	83427.90	8.42	1	–
	27	gi|222635113	LOC_Os06g09230	87846.50	8.41	1	2.60
	28	gi|218191002	LOC_Os02g35760	39122.50	8.15	0	–
	29	gi|115446775	LOC_Os02g35760	39051.40	8.15	0	–
	30	gi|115456539	LOC_Os03g62700	40775.30	6.15	0	–
	31	gi|115436274	LOC_Os01g21970	40275.10	9.35	0	0.31
	32	gi|218189537	LOC_Os01g67340	40499.20	6.38	0	–
	33	gi|222613313	LOC_Os10g42110	99869.10	6.08	0	7.11
	34	gi|125584817	LOC_Os03g04050	59402.40	6.01	0	–
SD	35	gi|297725777	LOC_Os07g36780	90414.30	6.71	2	–
URK	36	gi|222613001	LOC_Os10g33650	53049.80	9.75	1	–
not_assigned	37	gi|115466176	LOC_Os06g03970	109035.50	5.36	1	–

Statistical information were in Additional file 8

*TMD* transmembrane domain

### Transporters in pollen plasma membrane

To understand the mechanisms underlying ion and metabolite flux across the rice PM, we systemically identified transporters in the pollen PM proteome. The transporter classification (TC) system was used to build a transporter classification database (TCDB, http://www.tcdb.org.) with about 10,000 representative and putative non-redundant transporters. By blast searching this database, we identified 209 transporters (matched to 161 loci) in the rice pollen PM proteome; these transporters involved 33 families (Table 2, Additional file 12). These transporters are involved in exchanges and flux across the PM of diverse inorganic ion and metabolites such as Ca^2+^ (*n* = 12), H^+^ (*n* = 38), K^+^ (*n* = 22), Cl^−^ (*n* = 6), Mg^2+^ (*n* = 5), sugar (*n* = 24), phospholipids (*n* = 3), amino acid/oligopeptide (*n* = 16), phosphate (*n* = 7) and sulfate (*n* = 3) (Table 2). In total, 34 transporters showed changed abundance during pollen germination. Abundance-increased transporters were the H^+^ transporters gi|218184289, gi|194033213, gi|194033219 and gi|218199814), one K^+^ transporter (gi|125533127), 3 sugar transporters (gi|222636644, gi|115478530 and gi|108706417), one ABC transporter (gi|218188091), 2 phospholipid transporters (gi|40253457 and gi|53793271), 2 oligopeptide transporters (gi|215697740 and gi|90265689) and 3 other transporters (gi|338817657, gi|10140720 and gi|38567827), and abundance-decreased transporters were another 7 H^+^ transporters (gi|125597623, gi|115469362, gi|297597907, gi|115451943, gi|115444549, gi|115437984 and gi|115465801), 3 K^+^ transporters (gi|115462953, gi|15128390 and gi|297722665), one Mg^2+^ transporter (gi|115454637), 2 sugar transporters (gi|222622219 and gi|115434360), 3 ABC transporters (gi|115485837, gi|115477865 and gi|218198932) and 2 other transporters (gi|90399194 and gi|75253347) (Additional file 12).

**Table 2:** Two hundred nine transporters identified in plasma membrane of MPGs/GPGs in rice

Substrate	Family (TCDB)	No. of transporters/channels
Ca^2+^	TC 1.A.1-The Voltage-gated Ion Channel (VIC) Superfamily	3
	TC 3.A.3-The P-type ATPase (P-ATPase) Superfamily	9
H^+^	TC 3.A.2-The H^+^- or Na^+^-translocating F-type, V-type and A-type ATPase (F-ATPase) Superfamily	19
	TC 3.A.3-The P-type ATPase (P-ATPase) Superfamily	13
	TC 3.A.10-The H^+^, Na^+^-translocating Pyrophosphatase (M^+^-PPase) Family	3
	–	3
K^+^	TC 1.A.1-The Voltage-gated Ion Channel (VIC) Superfamily	2
	TC 2.A.37-The Monovalent Cation:Proton Antiporter-2 (CPA2) Family	10
	TC 2.A.72-The K^+^ Uptake Permease (KUP) Family	10
Cl^−^	TC 1.A.17-The Calcium-Dependent Chloride Channel (Ca-ClC) Family	1
	TC 1.B.8-The Mitochondrial and Plastid Porin (MPP) Family	3
	TC 2.A.49-The Chloride Carrier/Channel (ClC) Family	2
Mg^2+^	TC 1.A.35-The CorA Metal Ion Transporter (MIT) Family	5
Anion-selective	TC 1.A.23-The Small Conductance Mechanosensitive Ion Channel (MscS) Family	2
Sugar	TC 2.A.1-The Major Facilitator Superfamily (MFS)	17
	TC 2.A.2-The Glycoside-Pentoside-Hexuronide (GPH):Cation Symporter Family	3
	TC 2.A.84-The Chloroplast Maltose Exporter (MEX) Family	2
	TC 9.A.58-The Sweet; PQ-loop; Saliva; MtN3 (Sweet) Family	2
Lipid-soluble precursor	TC 3.A.1-The ATP-binding Cassette (ABC) Superfamily	26
	–	6
Boron	TC 2.A.31-The Anion Exchanger (AE) Family	2
Phospholipid	TC 3.A.3-The P-type ATPase (P-ATPase) Superfamily	3
Amino acid/oligopeptide	TC 1.B.30-The Plastid Outer Envelope Porin of 16 kDa (OEP16) Family	1
	TC 2.A.3-The Amino Acid-Polyamine-Organocation (APC) Superfamily	2
	TC 2.A.17-The Proton-dependent Oligopeptide Transporter (POT) Family	1
	TC 2.A.18-The Amino Acid/Auxin Permease (AAAP) Family	6
	TC 2.A.67-The Oligopeptide Transporter (OPT) Family	6
Water	TC 1.A.8-The Major Intrinsic Protein (MIP) Family	1
Phosphate	TC 2.A.1-The Major Facilitator Superfamily (MFS)	2
	TC 2.A.7-The Drug/Metabolite Transporter (DMT) Superfamily	1
	TC 2.A.29-The Mitochondrial Carrier (MC) Family	4
Sulfate	TC 2.A.53-The Sulfate Permease (SulP) Family	3
Micronutrient	TC 2.A.4-The Cation Diffusion Facilitator (CDF) Family	1
	TC 2.A.5-The Zinc (Zn^2+^)-Iron (Fe^2+^) Permease (ZIP) Family	1
	TC 3.A.3-The P-type ATPase (P-ATPase) Superfamily	2
	TC 3.A.19-The TMS Recognition/Insertion Complex (TRC) Family	4
Others	TC 1.A.11-The Ammonia Channel Transporter (Amt) Family	1
	TC 2.A.1-The Major Facilitator Superfamily (MFS)	3
	TC 2.A.7-The Drug/Metabolite Transporter (DMT) Superfamily	4
	TC 2.A.47-The Divalent Anion:Na + Symporter (DASS) Family	1
	TC 3.A.5-The General Secretory Pathway (Sec) Family	2
	TC 3.A.9-The Chloroplast Envelope Protein Translocase (CEPT or Tic-Toc) Family	4
	–	13

“_” means no classification in TCDB

### Comparison between rice pollen PM proteome and transcriptome

To evaluate the possible relation of PMrPs and their transcripts, we retrieved previously reported data for transcripts expressed in MPGs and GPGs [30]. The analysis involved 5,939 transcripts detected in MPGs and 5,945 in GPGs, for a total of 7,161 unique transcripts expressed in MPGs or/and GPGs.

**Fig. 5:**
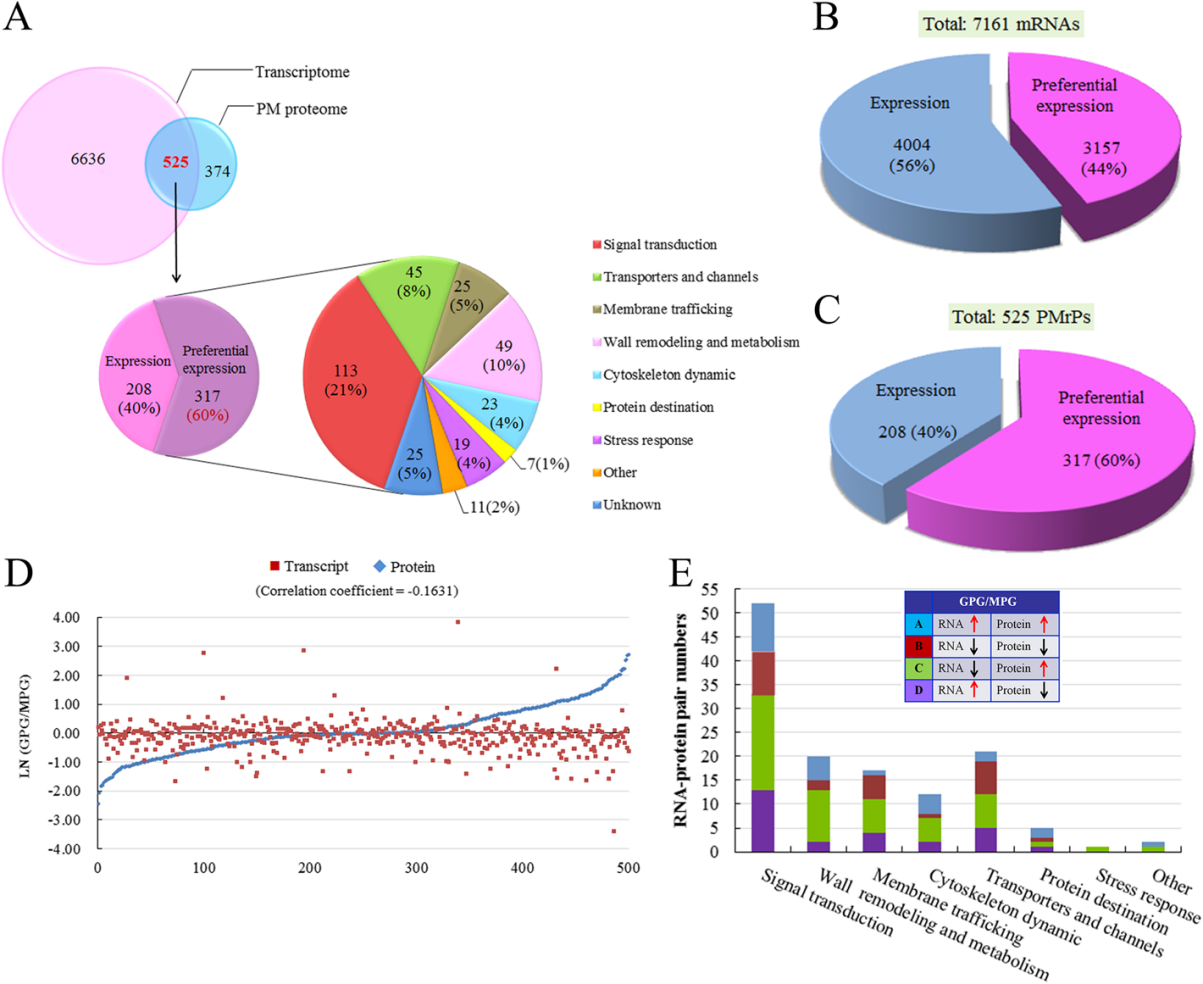
Comparison of pollen PM protein and transcript levels. **a** 525 of the PMrPs have corresponding transcripts, and 317 of these transcripts (60%) are pollen-preferential and are mainly implicated in signal transduction. **b** 44% of the pollen mRNA are pollen-preferential. **c** 60% of the PMrPs transcripts are pollen-preferential. **d** No significant correlation between PMrP expression and their transcript expression. **e** Four expression patterns of PMrP–transcript pairs and their distribution in each functional category

Locus number comparison showed 525/899 pollen PMrPs (58.4%) with corresponding transcripts; 317 of these were pollen-preferential (cutoff at *Ratio ≥ 2.0*, Ratio = *MAX (median (MPGs), median (GPGs))/MAX (callus cells1-3, roots1-3, leaves1-3)*) (Additional file 13) and were mainly involved in signal transduction, wall remodeling and metabolism and transporters (Fig. 5a). Unexpectedly, 374/899 of the pollen PMrPs (41.6%) had no corresponding transcripts in the dataset of 7,161 transcripts. Their transcripts may be short-lived or extremely low-abundant or the protein was synthesized at early stages of pollen development and deposited for late requirement [31]. We found that 60% of transcripts for encoding pollen PMrPs were pollen-preferential, with only 44% of total MPG/GPG-expressed transcripts being pollen-preferential (Fig. 5b and c), which suggests that PMrP-encoding genes have high organ- or cell-specific expression.

Next, we analyzed the correlation of expression profiles between pollen PMrPs and their transcripts. In total, 500 of the 525 pollen PMrP–transcript pairs had quantitative information for both protein and RNA levels (Additional file 14). The expression profiles of PMrPs and their transcripts were not significantly correlated (correlation coefficient −0.1631) (Fig. 5d). When this analysis was restricted to the 192 differentially expressed PMrPs, 130 of which had corresponding transcripts, the 130 protein–transcript pairs were assigned to 4 patterns: (A) both mRNA and protein levels increased in GPGs (25 pairs), (B) both mRNA and protein levels decreased in GPGs (25 pairs), (C) mRNA levels decreased and protein levels increased in GPGs (53 pairs), and (D) mRNA levels increased and protein levels decreased in GPGs (27 pairs) (Fig. 5e, Additional file 14). In total, 74% of these differentially expressed PMrP showed discordant expression with their mRNAs. Thus, the protein expression pattern of PMrPs was not closely related to their mRNA levels omic-wide, which indicates the importance of PM proteomic studies in understanding pollen function.

## Discussion

We prepared high-purity pollen PM by using the aqueous polymer two-phase system and alkali buffer treatment and identified PMrPs and differentially expressed PMrPs between mature and germinated pollen by using LC-MS and iTRAQ-based quantitative proteomics approaches. This study revealed dynamic characteristics of the pollen PM proteome and a large set of RLKs and transporters in the proteome. As well, the expression pattern of PMrPs was in general inconsistent with that of corresponding mRNAs.

### Pollen PM-related protein-encoding genes show biased distribution in rice chromosomes

Studies have shown that 26% of genes of the human genome [32] and 25% of genes of the *Arabidopsis* genome [33] encode membrane proteins. Accordingly, the rice genome, with an estimated 50,000-60,000 genes [34, 35], encodes about 13,000 membrane proteins. However, PM protein-encoding genes possibly represent a small percentage of a genome. Proteomic clues suggest that only 3% genes in a genome encode PM proteins [36], for about 750 PM proteins in *Arabidopsis* [37] and more than 2,500 PM proteins in humans [38]. Therefore, rice may contain about 1,500 PM proteins. Our study revealed 1,121 PMrPs (matched 899 loci) in rice pollen, which should represent nearly 60% of the predicted PM proteins.

Data analysis showed that annotated genes appear to distribute in rice chromosomes at a similar frequency, with the exception of chr 1, which has a larger number of genes than other chromosomes. However, the distribution frequency of PMrPs and differentially expressed PMrPs was significantly higher than that of genome genes in chr 1, 2 and 3, with the highest biased distribution for differentially expressed PMrPs in chr 3 (Fig. 3c). As well, these enriched PMrPs and differentially expressed PMrPs in these chromosomes showed significantly functional skew toward signal transduction (Additional file 9). Thus, the biased distribution may reflect a mechanism to coordinate these diverse components of a pathway in action, although further studies are needed to address the phenomena and the biological roles of the biased distribution of pollen PMrPs.

### Pollen synthesizes a set of PM proteins specialized for pollen function

Pollen grains of most plants are metabolically quiescent when released from anthers and are tolerant of desiccation for spreading over long distances by pollinators. On loading onto the stigma, pollen germinates to generate a fast-growing PT, which journeys within the pistil via turgor-driving growth at the tip. Thus, PT growth and successful fertilization requires active membrane and wall material transportation, wall remodeling, and signal and materials communication across the PM [39].

The pollen proteome has highly represented wall remodeling and metabolism-related proteins, which differs from vegetative cell proteomes [40]. Consistent with previous findings, pollen PM-related wall remodeling and metabolism proteins were preferentially represented in both pollen PMrP and differentially expressed PMrP sets in our study. Such proteins are involved in wall synthesis, loosening and extension. For example, we revealed abundance-increased enzymes for pectin biosynthesis, such as methylesterase (PME) (gi|115452515, gi|115452623, gi|125601598, gi|115486641, and gi|108864650), and abundance-decreased enzymes for pectin degradation, such as glycosyl hydrolases (gi|115443693), PME inhibiter (gi|297721723), and polygalacturonase (gi|115437052 and gi|125555670) (Additional file 8). This finding is compatible with the importance of pectin in PT growth.

Along with the highly represented wall remodeling and metabolism PMrPs, those implicated in signal transduction, transporters and membrane trafficking were overrepresented in both pollen PMrP and differentially expressed PMrP sets. These membrane trafficking-associated PMrPs mainly involve exocytosis and endocytosis pathways, vesicle targeting and vesicle-mediated transport, which sustain the material basis for cell-wall and PM extension and thus support fast PT growth [41]. We revealed a large set of PMrPs for diverse signaling pathways such as auxin, ABA, calcium, phospholipid, and GTPase signaling; mitogen-activated protein kinase phosphorylation cascades; and receptor-like kinase pathways, which suggest that pollen function requires multiple signaling pathways and coordination of these pathways. We also revealed multiple transporters involved in flux of diverse ions and metabolites across the PM, which are consistent with the importance of the ion gradient and dynamics in PT growth and integrity maintenance (for details, see below). Together, these results indicate that these highly represented terms are compatible with the requirement of unique PT growth mechanism and function, thus providing insights into the molecular network for PT growth and function.

Previous proteome and transcriptome results showed mature and germinated pollen had similar protein and mRNA profiles [30, 40]. Thus, germination and early PT growth depend mainly on presynthesized mRNAs and proteins. Yet the impact of mRNA levels on protein expression is unclear. Here, we evaluated the association between variations in mRNA and protein expression levels. The protein expression patterns of PMrPs and their mRNA levels were not significantly correlated, with uncorrelated mRNA expression profiles for 74% of these differentially expressed PMrPs. This finding differs from the empirical conclusion that the correlation between protein and mRNA levels is generally modest [42]. Studies of mammals have revealed that protein levels are heritable molecular phenotypes and evolve under greater evolutionary constraints than mRNA levels [42, 43]. This highly discordant expression between PMrP protein and mRNA levels suggests that pollen has mechanisms to build a PM proteome for fast tip-growth and function, although further studies are needed to understand the mechanisms.

### Pollen has multiple types of PM-localized RLKs

Fast-growing PTs within pistils need to constantly monitor the surface tension of the cell wall and PM and communicate with female stimuli to coordinate PT growth, integrity and timely growth arrest and rupture as they enter the ovule, but the molecular mechanisms remain largely unknown. RLKs relay extracellular and cell surface signals for initiating intracellular signaling cascades, thereby representing the central components of the signaling pathways mediating cell–cell interaction [1]. Transcriptome studies revealed 43 and 99 RLK genes expressed in *Arabidopsis* MPGs and rice GPGs/MPGs [4, 30], respectively, about 7 and 11% of the predicted RLK genes in the respective genomes (Additional file 11). Here, we revealed the protein levels of 37 RLKs from 8 subfamilies in rice pollen, with 6 levels changed with pollen germination (Table 1).

**Fig. 6:**
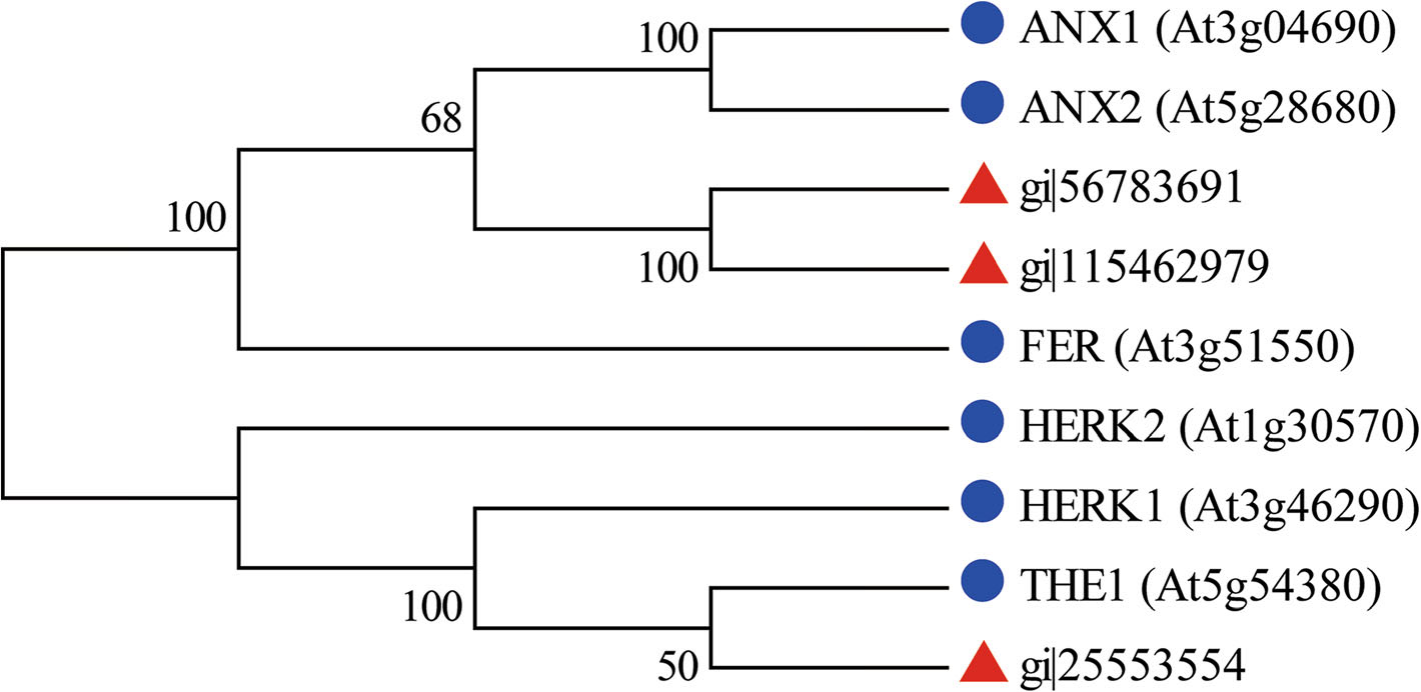
Phylogenetic relationship of the identified CrRLK1Ls in rice pollen and functionally known *Arabidopsis* CrRLK1Ls. The tree was created by using MEGA 4.0

CrRLK1Ls have putative carbohydrate-binding domains, the malectin domain, in their extracellular regions and are considered candidates for sensing changes in the cell surface or external stimuli [44]. In *Arabidopsis*, THE1, HERK1 and HERK2 expressed in vegetative tissues are required for cell extension [45]. Pollen-expressed ANX1 and ANX2 are redundant for regulating PT integrity [3, 4], whereas synergid-expressed FER regulates PT reception and timely rupture [46]. We revealed three CrRLK1Ls (gi|56783691, gi|115462979 and gi|25553554) in rice pollen, the latter showing increased expression in germinated pollen (Table 1). Sequence alignment showed that they had a variable malectin domain but highly conserved kinase domains with THE1, HERK1, HERK2, ANX1, ANX2 and FER (Additional file 15). gi|56783691 and gi|115462979 are phylogenetically close to ANX1 and ANX2, but gi|25553554 is phylogenetically close to THE1 (Fig. 6). gi|25553554, termed RUPO, regulates PT integrity by interacting with K^+^ transporters [6]. These results suggest that multiple CrRLK1L signaling pathways orchestrate PT growth and cross-talk between PTs and female cells.

**Fig. 7:**
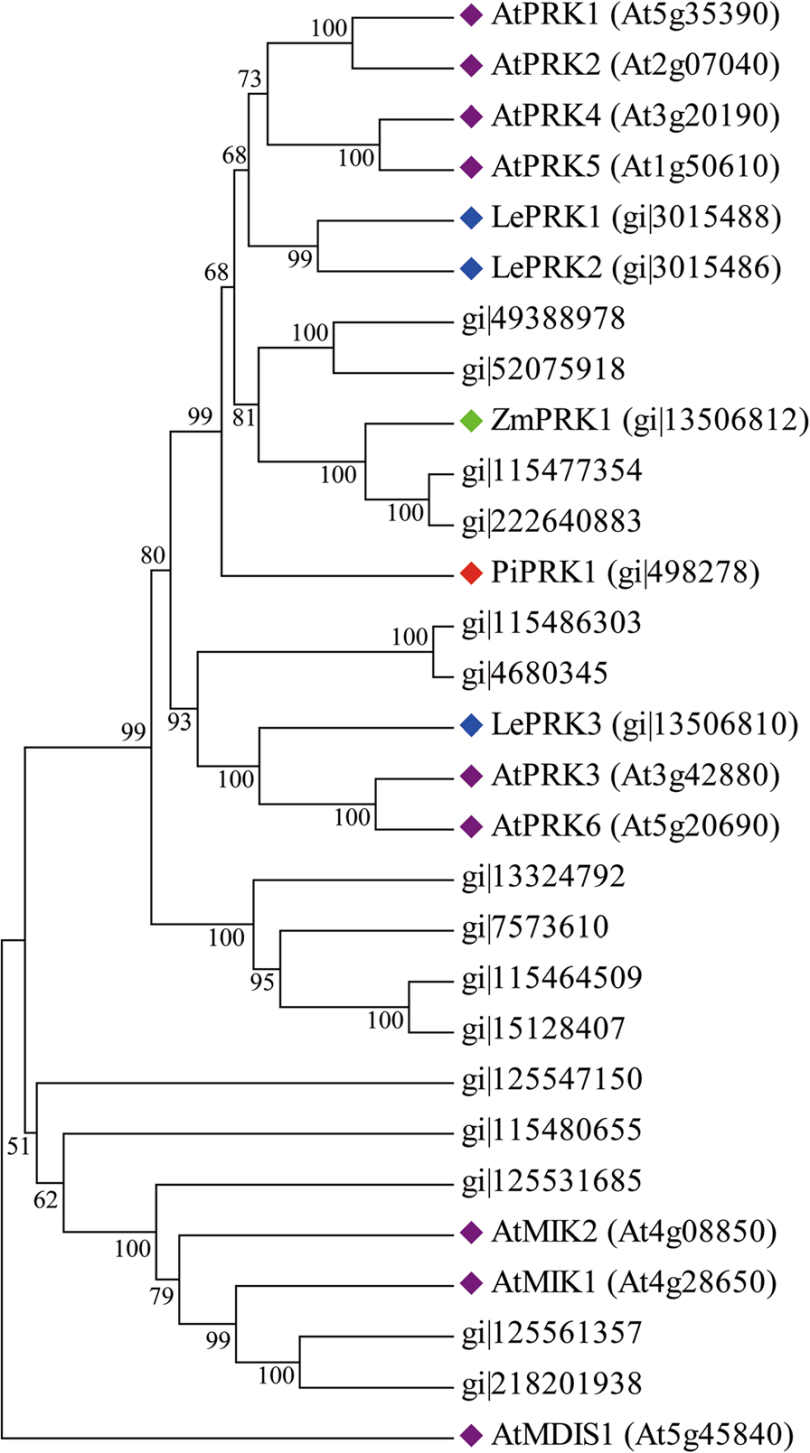
Phylogenetic relationship of identified LRR-RLKs in rice pollen and functionally known members of this subfamily. This analysis involves 15 LRR-RLKs identified in rice pollen PM and 14 functionally known members from *Arabidopsis thaliana* (At), *Petunia inflate (*Pi), *Lycopersicon esculentum* (Le), and *Zea mays* (Zm). The tree was made by MEGA 4.0

The LRR-RLK subfamily contains the most members of RLKs and has 384 members in rice (312 by RGAP7.0) [29]. We revealed 15 LRR-RLKs in rice pollen PM (Table 1). To obtain functional features, we analyzed their phylogenetic relations with functionally known pollen-specific LRR-RLKs (PRKs) [47] required for pollen maturation (PiPRK1 from *Petunia inflate*) [48], pollen–pistil interaction (LePRK1 and LePRK2 from *Lycopersicon esculentum*) [28], PT elongation (AtPRK2 from *Arabidopsis thaliana*) [49] and PT targeting (AtPRK6, AtMDIS1, AtMIK1 and AtMIK2 from *Arabidopsis thaliana*) [8, 9]. Four of the 15 identified LRR-RLKs (gi|49388978, gi|52075918, gi|115477354 and gi|222640883) are in a clade with ZmPRK1 from *Zea mays*; in evolution, these LRR-RLKs appeared to differentiate from the dicot members AtPRP1-2 and 4–5, LePRK1-2 and PiPRK1 before the monocot–dicot split*.* gi|115486303 and gi|4680345 are in a clade with LePRK3, AtPRK3 and AtPRK6 and gi|125547150, gi|115480655, gi|125531685, gi|125561357 and gi|218201938 are in a clade with AtMIK1 and 2, which indicates their differentiation after the monocot–dicot split. The remaining 4 are in an independent clade without functionally known dicot homologs (Fig. 7). In fact, recent study revealed the pollen-expressed *Arabidopsis* LRR-RLKs MDIS1 and MIK acting as a pollen PM-localized, heteromer receptor for sensing egg-expressed attractant LURE1 to control PT reception and guidance [8]. AtPRK6 was found to be another receptor of the species-specific AtLURE1 that can trigger the Rop signaling cascade to regulate PT growth [9]. Together, these findings suggest that multiple male receptors can respond to the same female attractor within the same species.

RLCK is another large subfamily of RLKs that lack the transmembrane domain [29]. Two pollen-preferential RLCKs in *Arabidopsis* – LIP1 and LIP2 – are anchored to the PM of the PT tip via palmitoylation, and they can perceive the female signal – LURE1 – attraction [50]. SD-RLKs have the extracellular domains homologous to the secreted *S*-locus glycoproteins (SLGs) that display genotype-specific sequence polymorphisms. *S* receptor kinases were proposed to be responsible for the allelic specific self-incompatibility response along with SLGs [51]. We identified 13 RLCKs and one SD-RLK, with gi|222635113 and gi|222613313 increased and gi|115436274 decreased in levels in GPGs (Table 1), as candidates for pollen–pistil recognition. In addition, we revealed the RLKs Extensin, PERK and URK subfamilies, although their functions in PT–pistil interaction remain unknown. Together, our results indicate that multiple RLK signaling pathways are required for orchestrating PT growth and cross-talk between PTs and female cells and provide important candidates for further understanding the mechanisms.

### Coordination of diverse ion and metabolites across the pollen PM

The gradient and homeostasis of Ca^2+^, H^+^, K^+^ and Cl^−^ are critical for pollen germination and PT growth [52], but the molecular mechanisms underlying the gradient and homeostasis are not fully understood. We revealed 209 transporters in rice pollen PM involved in flux across the PM of Ca^2+^, K^+^, H^+^, Cl^−^, water, sugar, amino acids, oligopeptides and phospholipids (Table 2). Tip-fused Ca^2+^ gradient is the central player orchestrating the cross-talk of diverse signaling pathways and the ion is also important for regulating the differential plasticity of the PT apical and subapical wall [53]. Lines of evidence have shown that in *Arabidopsis*, transporters for Ca^2+^ flux were involved in PT growth, PT–synergid contact [14] and PT guidance [16]. Here, we showed 12 Ca^2+^ transporters in rice pollen PM from the VIC and P-ATPase superfamily (Table 2), which indicates their importance in controlling Ca^2+^ gradient and homeostasis in pollen.

The increased pH from the apical to the subapical region functions as an important node in regulating diverse activities in PT, such as the wall plasticity, vesicle transport and actin dynamics [54]. The pH gradient is mainly mediated by PM H^+^-ATPases (PMAs) that promote H^+^ exflux in the subapical region [55]. In rice pollen PM, we revealed 12 PMAs from the P-ATPase superfamily (Table 2), which highlights their central roles in sustaining PT alkalinity. Which transporters are responsible for H^+^ influx in the PT apex are unknown, but the nonspecific cation channels are generally considered responsible for apical H^+^ influx [52]. The rice pollen PM showed a novel cation-transporting ATPase (OsP5, gi|218196773) of the P-type ATPase superfamily, which gives some hints for PT H^+^ influx. Besides the PMAs, 13 VHAs and 3 H^+^-PPases were revealed, with the VHA subunit H (gi|218199814) significantly increased and subunit A (gi|115444549) and C (gi|115437984 and gi|115465801) decreased in levels in GPGs; they may also affect PT growth by sustaining cytosolic pH homeostasis [54].

K^+^ is an essential mineral for pollen germination and PT growth [56]. Studies have demonstrated that K^+^ channels were required for PT growth [17] and directional growth to the ovule in *Arabidopsis* [18] and integrity in maize [19]. We found 22 cation or specific K^+^ transporters (Table 2), which suggests that multiple transporters are involved in maintaining K^+^ homeostasis and PT turgor pressure. In addition, studies showed that PTs have a tip-fused negative Cl^−^ gradient correlated with vesicle movement and close relationship with water homeostasis [57]. Besides the functionally known anion channel SLAH3, which is activated by calcium-dependent protein kinases via phosphorylation for Cl^−^ exflux [58], we identified 3 anion channels and 3 Cl^−^ channels in rice pollen PM (Table 2), thus supplying the molecular information for insights into the Cl^−^ gradient.

Moreover, we revealed 5 Mg^2+^ transporters and 2 mechanosensitive channels MscS-like in pollen PM (Table 2). MscS-like 8 plays key roles in helping *Arabidopsis* pollen survive hypo-osmotic shock during rehydration [59]. Sucrose transporters (SUTs) and monosaccharide transporters (MSTs) are suggested to participate in pollen development, germination and PT growth but show high functional redundancy [60, 61]. The rice genome has 5 SUT genes (*OsSUT1-5*); only *OsSUT1* and *OsSUT3* transcripts were detectable in pollen [62]. We identified both OsSUT1 (gi|108706417) and 2 OsSUT3 isoforms (gi|222641552 and gi|115481924) in pollen PM (Additional file 12), which agrees with their functions in pollen germination and starch accumulation [62]. The rice genome has 65 predicted MST genes [63], but so far only 7 have been characterized [64]. We identified 15 MSTs from STP, pGlcT, AZT, INT, PLT, and ERD subfamilies in the PM, with 2 (gi|222636644, gi|115478530) increased and 2 (gi|222622219 and gi|115434360) decreased in levels in GPGs (Additional files 8 and 12 ). Several ATP-binding cassette (ABC) transporters were found to function in exine lipid-soluble precursor secretion [65]. ABC transporters consist of 2 nucleotide-binding domains (NBDs) to bind and hydrolyse ATP and 2 transmembrane domains involved in substrate recognition. According to the NBD evolutionary relationship, the transporters can be classified into 8 major subfamilies (A-G and I) [66]. ABCG transporters were well known to be expressed in tapetum, but little was known about whether they were expressed in pollen [67]. We first identified 6 ABCG transporters and also found 23 other ABC transporters in the PM (Additional file 12). We have little knowledge about the substrates and molecular functions of the 29 ABC transporters.

**Fig. 8:**
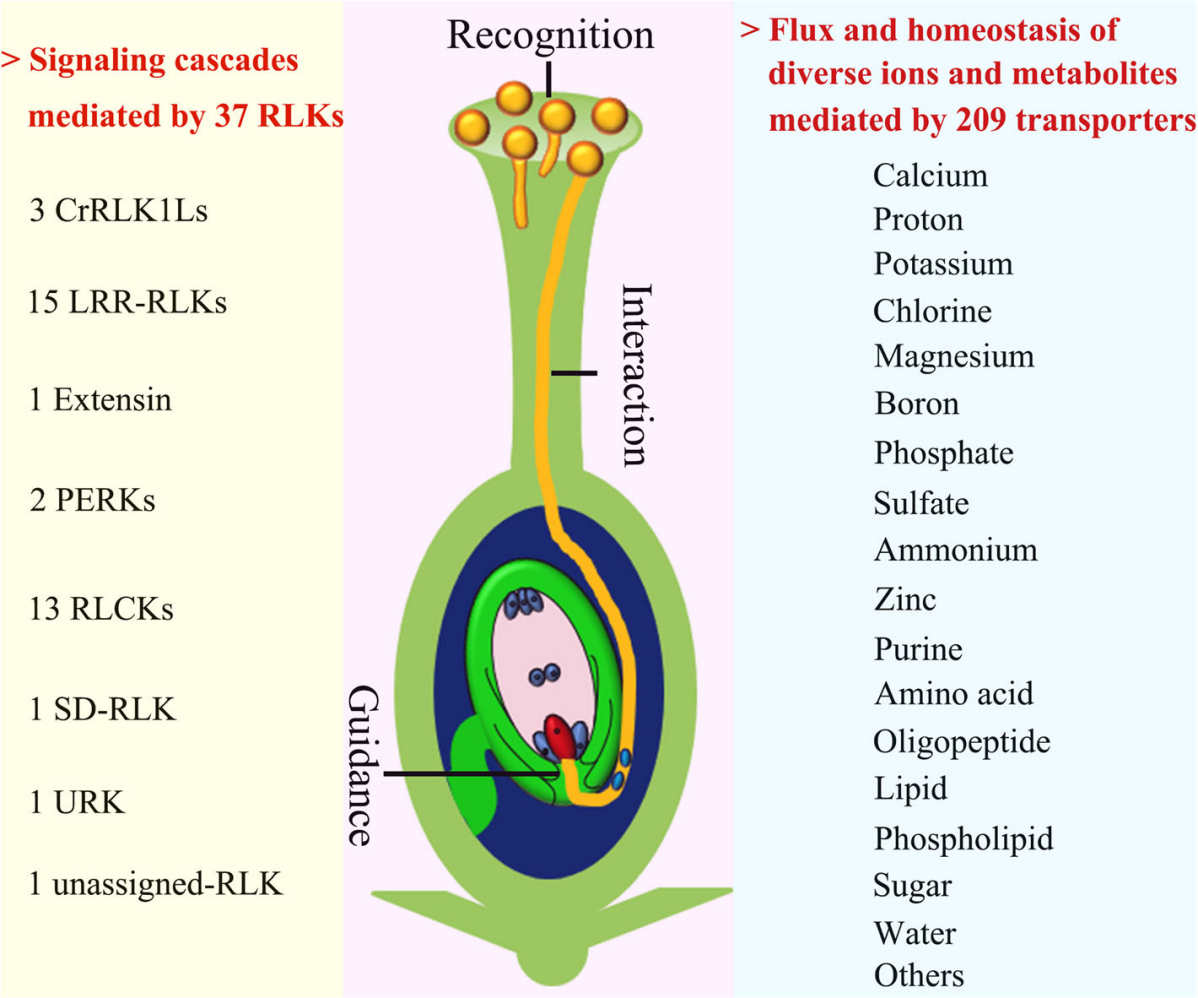
Diversified RLKs and transporters are fine-tuned and coordinated in regulating pollen-pistil interaction and pollen tubes tip growth

Finally, we identified other transporters in the rice PM (Additional file 12): (1) the pollen-specific boron efflux transporter OsBOR4, implicated in pollen germination and PT elongation [68]; (2) 3 P-type ATPases belonging to the aminophospholipid flipping (ALA) subfamily [69], which may be involved in establishing membrane lipid asymmetry [70]; (3) 9 amino acid transporters and 7 oligopeptide transporters (The types of transporters display different substrate specificities and may help pollen to sustain the rehydration process by supplying compatible solutes [71]); (4) an aquaporin that may adjust the water condition in pollen; and (5) 7 phosphate, 3 sulfate, 8 micronutrient and some other transporters for various substrates, which may provide the essential nutrients to sustain pollen activity under unclear mechanisms. Together, the large set of diversified transporters identified in pollen PM suggests that fine-tuned multiple ion and metabolite homeostasis and coordination of homeostasis are crucial for PT growth and function. Thus, we provide important molecular information for understanding the mechanisms underlying the homeostasis and coordination (Fig. 8).

## Conclusions

We revealed 1,121 PMrPs and 192 differentially expressed PMrPs during rice pollen germination. Pollen presynthesizes a set of PM proteins for PT growth and interaction with pistils. The pollen PM protein levels are in general discordant with their mRNA levels, and their genes showed biased distribution in rice chromosomes. The pollen PM have a large set of RLKs mediating cell–cell interaction and transporters for flux of diversified ions and metabolites (Fig. 8). This study provides a resource to further dissect the molecular mechanisms by which pollen or the PT controls PMrP abundance and monitors interactions and ion and metabolite exchanges with female cells in rice.

## Additional files

Additional file 1:
*Rapi*Gest-assisted PM protein digestion. Proteins of carbonate-washed PM proteins treated without (A, B) or with trypsin coupled with 0.2% *Rapi*Gest (C, D) were separated by 10% SDS-PAGE and stained with Coomassie brilliant blue. MPG, mature pollen grain; GPG, germinated pollen grain. 20 μg loaded per lane. (PDF 1401 kb)
Additional file 2: Separation of iTRAQ-labeled peptides through SCX chromatography. The peptides from experiments 1 and 2 were fractionized by strong cation exchange chromatography (SCX) independently. After eluted by ammonium chloride, both of them were recombined into 16 fractions according to the 214 nm UV absorbance signal. (PDF 2055 kb)
Additional file 3: Summary of spectra, peptides, and proteins identified in the 2 independent iTRAQ experiments. (PDF 11 kb)
Additional file 4: All proteins identified in the experiments and their quantitative information. (XLSX 590 kb)
Additional file 5: Plasma membrane-related proteins. (XLSX 240 kb)
Additional file 6: Pearson analysis shows high quantitative reproducibility between the two independent iTRAQ experiments. ln [Exp 1], ln-transformed protein expression change ratio from experiment 1 (GPG/MPG = 117/115); ln [Exp 2], ln-transformed protein expression change ratio from experiment 2 (GPG/MPG = 115/117). (PDF 866 kb)
Additional file 7: Mass spectrometry spectra show the repeatability of iTRAQ quantitative information. (PDF 634 kb)
Additional file 8: Differentially expressed plasma membrane-related proteins. (XLSX 56 kb)
Additional file 9: PMrPs and differentially expressed PMrPs show biased distribution in chromosomes 1, 2 and 3. A, PMrPs of each functional group distributed in rice 12 chromosomes. B, differentially expressed PMrPs of each functional group distributed in rice 12 chromosomes. (PDF 3061 kb)
Additional file 10: Western blot validation of the iTRAQ quantitative information. (PDF 94 kb)
Additional file 11: Summary of receptor-like kinases (RLKs) in *Arabidopsis thaliana* and *Oryza sativa*. (XLSX 26 kb)
Additional file 12: Classification of 209 transporters identified from rice MPG and GPG plasma membrane. (XLSX 39 kb)
Additional file 13: Qualitative comparison of rice pollen transcriptome and proteome. (XLSX 204 kb)
Additional file 14: Quantitative comparison of pollen transcriptome and proteome. (XLSX 76 kb)
Additional file 15: Multiple sequence alignment of CrRLK1Ls. Sequence alignments of identified rice pollen CrRLK1Ls gi|56783691, gi|115462979 and gi|25553554, and functionally known HERK1 (AT3G46290), HERK2 (AT1G30570), THE1 (AT5G54380), ANX1 (AT3G04690), ANX2 (AT5G28680) and FER (AT3G51550) from Arabidopsis. These CrRLK1Ls have a variable malectin domain (underlined in purple) and highly conserved kinase domain (in red) along with one transmembrane domain (in orange) predicted by SMART. Identical and similar amino acids are shaded in blue and yellow. (PDF 18045 kb)

## Abbreviations

CrRLK1L: *Catharanthus roseus* RLK1-like
GPGs: Germinated pollen grains
iTRAQ: Isobaric tags for relative and absolute quantitation
LRR: Leucine-rich repeat
MPGs: Mature pollen grains
PM: Plasma membrane
PMrPs: PM-related proteins
PT: Pollen tube
RLKs: Receptor-like kinases
VC: Vegetative cell
