# Supplementary figures and images for: Comparative proteomic analysis reveals a dynamic pollen plasma membrane protein map and the membrane landscape of receptor-like kinases and transporters important for pollen tube growth and interaction with pistils in rice
Source: BMC Plant Biol. 2017 Jan 5;17:2. doi: 10.1186/s12870-016-0961-7 (PMC5217431; doi:10.1186/s12870-016-0961-7)

# Proteins

# Peptides

MPG GPG

MPG GPG marker

kDa

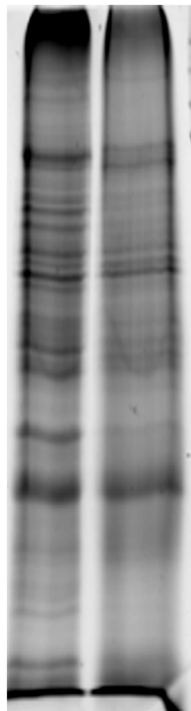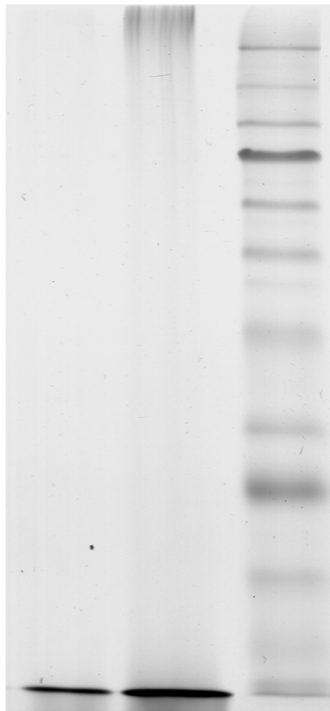

230

150

100

80

60

50

40

30

25

20

15

10

A

B

C

D

Supplement: Additional file 1: — RapiGest-assisted PM protein digestion. Proteins of carbonate-washed PM proteins treated without (A, B) or with trypsin coupled with 0.2% RapiGest (C, D) were separated by 10% SDS-PAGE and stained with Coomassie brilliant blue. MPG, mature pollen grain; GPG, germinated pollen grain. 20 μg loaded per lane. (PDF 1401 kb) [file 12870_2016_961_MOESM1_ESM.pdf]

## Experiment 1

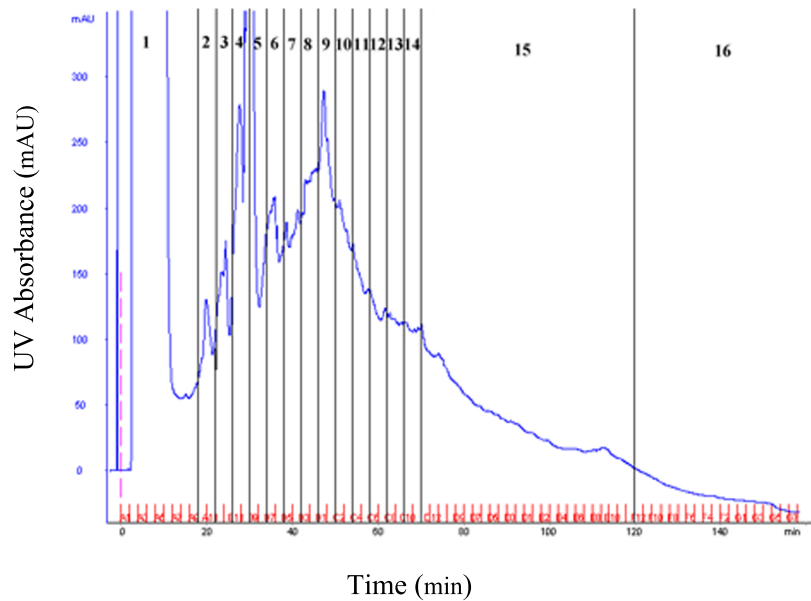

## Experiment 2

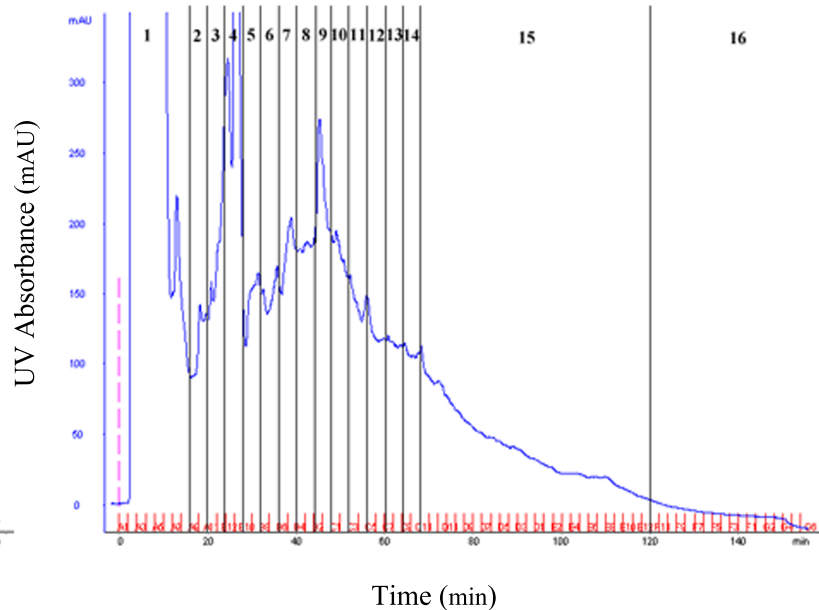

Supplement: Additional file 2: — Separation of iTRAQ-labeled peptides through SCX chromatography. The peptides from experiments 1 and 2 were fractionized by strong cation exchange chromatography (SCX) independently. After eluted by ammonium chloride, both of them were recombined into 16 fractions according to the 214 nm UV absorbance signal. (PDF 2055 kb) [file 12870_2016_961_MOESM2_ESM.pdf]

**Pearson's Correlation=0.877**

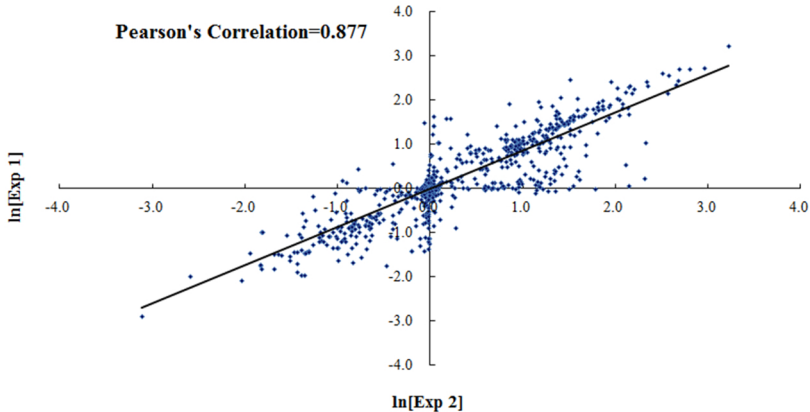

Supplement: Additional file 6: — Pearson analysis shows high quantitative reproducibility between the two independent iTRAQ experiments. ln [Exp 1], ln-transformed protein expression change ratio from experiment 1 (GPG/MPG = 117/115); ln [Exp 2], ln-transformed protein expression change ratio from experiment 2 (GPG/MPG = 115/117). (PDF 866 kb) [file 12870_2016_961_MOESM6_ESM.pdf]

A

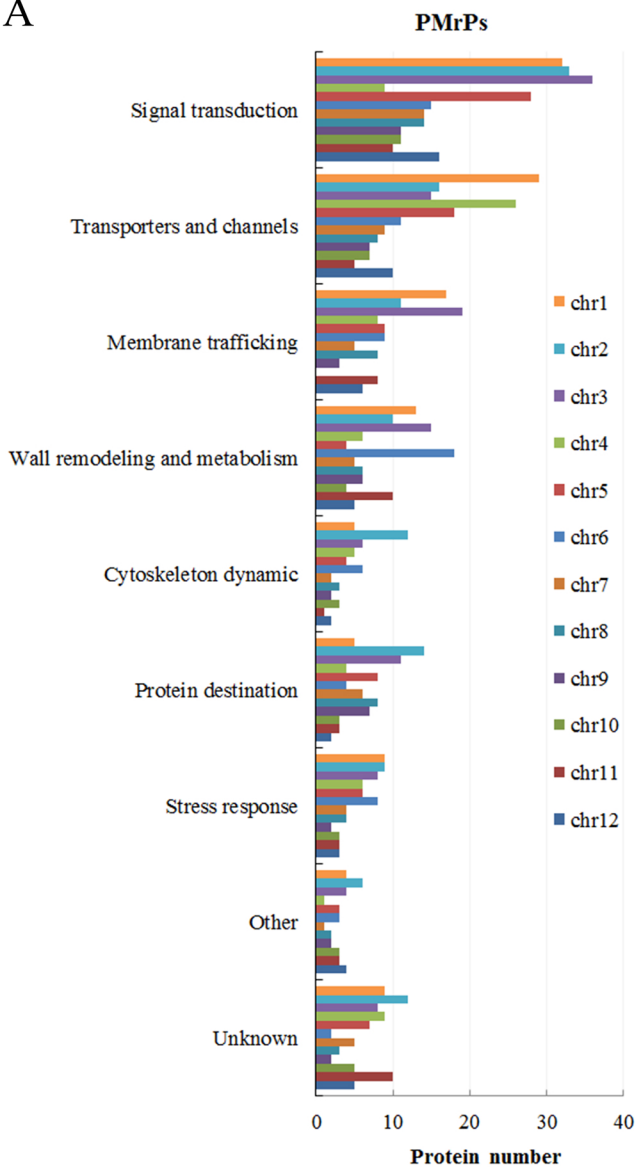

B

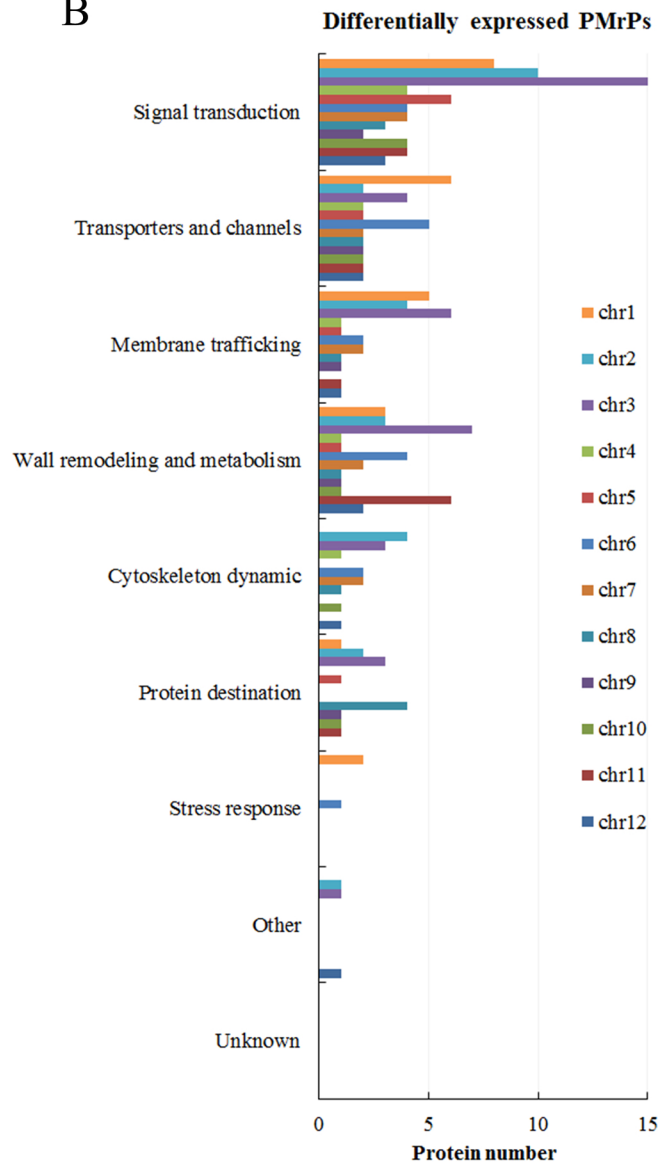

Supplement: Additional file 9: — PMrPs and differentially expressed PMrPs show biased distribution in chromosomes 1, 2 and 3. A, PMrPs of each functional group distributed in rice 12 chromosomes. B, differentially expressed PMrPs of each functional group distributed in rice 12 chromosomes. (PDF 3061 kb) [file 12870_2016_961_MOESM9_ESM.pdf]
