# Supplementary material for: Comparative proteomic analysis reveals a dynamic pollen plasma membrane protein map and the membrane landscape of receptor-like kinases and transporters important for pollen tube growth and interaction with pistils in rice
Source: BMC Plant Biol. 2017 Jan 5;17:2. doi: 10.1186/s12870-016-0961-7 (PMC5217431; doi:10.1186/s12870-016-0961-7)
Supplement: Additional file 3: — Summary of spectra, peptides, and proteins identified in the 2 independent iTRAQ experiments. (PDF 11 kb) [file 12870_2016_961_MOESM3_ESM.pdf]

Additional file 3. Summary of spectra, peptides, and proteins identified in the 2 independent iTRAQ experiments

	**Total spectra**	**Identified peptides** (Confidence = 95%)	**Identified proteins** (**Decoy,** unused > 1.3, Two or more peptides matched)	**Identified proteins** (**normal**, unused > 1.3, Two or more peptides matched)	**Quantified proteins** (unused > 1.3, Two or more peptides matched)	**FDR (%)**	**Total identified proteins**
**Experiment 1**	109,114	24,256	1	1,473	1,381	0.07	1,979
**Experiment 2**	78,037	19,965	1	1,283	1,147	0.08	
