# Supplementary material for: Comparative proteomic analysis reveals a dynamic pollen plasma membrane protein map and the membrane landscape of receptor-like kinases and transporters important for pollen tube growth and interaction with pistils in rice
Source: BMC Plant Biol. 2017 Jan 5;17:2. doi: 10.1186/s12870-016-0961-7 (PMC5217431; doi:10.1186/s12870-016-0961-7)

Peptide Quantitation Information

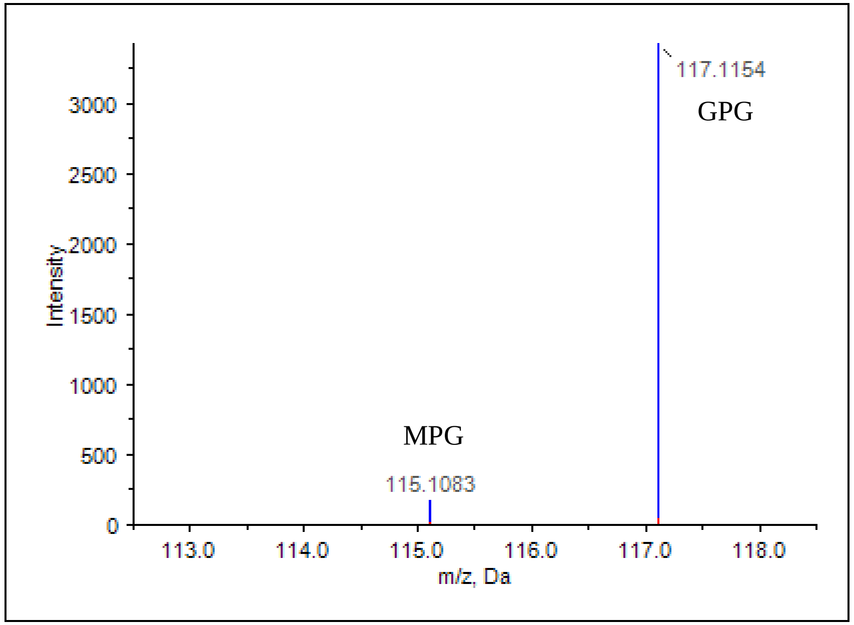

Precursor MS Region

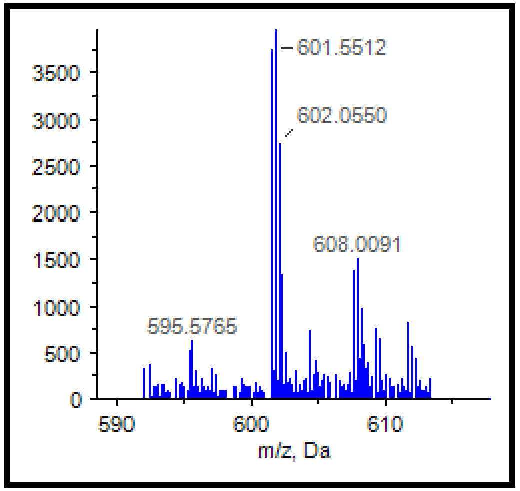

Accession: gi|115454901

Sequence:

QAFDDAIAELDTLGEESYK

Repeat 1: experiment 1

GPG (117) / MPG (115) = 8.55

Precursor mass (m/z): 601.55

Fragmentation Evidence for Peptide

IT4 QAFDDAIAELDTLGEESYK[IT4]

Residue	b	b+2	y	y+2
Q	273.1679	137.0876	2403.1806	1202.0939
A	344.2050	172.6062	2131.0199	1066.0136
F	491.2734	246.1404	2059.9828	1030.4950
D	606.3004	303.6538	1912.9144	956.9608
D	721.3273	361.1673	1797.8875	899.4474
A	792.3644	396.6859	1682.8605	841.9339
I	905.4485	453.2279	1611.8234	806.4153
A	976.4856	488.7464	1498.7393	749.8733
E	1105.5282	553.2677	1427.7022	714.3547
L	1218.6123	609.8098	1298.6596	649.8334
D	1333.6392	667.3232	1185.5756	593.2914
T	1434.6869	717.8471	1070.5486	535.7779
L	1547.7710	774.3891	969.5009	485.2541
G	1604.7924	802.8999	856.4169	428.7121
E	1733.8350	867.4211	799.3954	400.2013
E	1862.8776	931.9424	670.3528	335.6800
S	1949.9096	975.4585	541.3102	271.1588
Y	2112.9730	1056.9901	454.2782	227.6427
K[IT4]	2385.1700	1193.0886	291.2149	146.1111

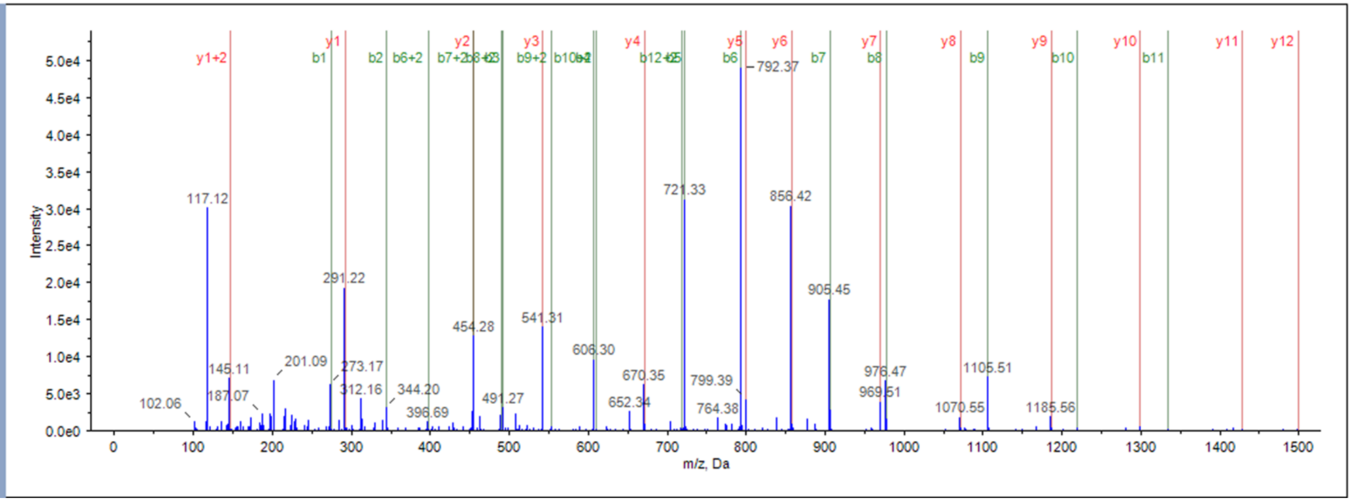

Peptide Quantitation Information

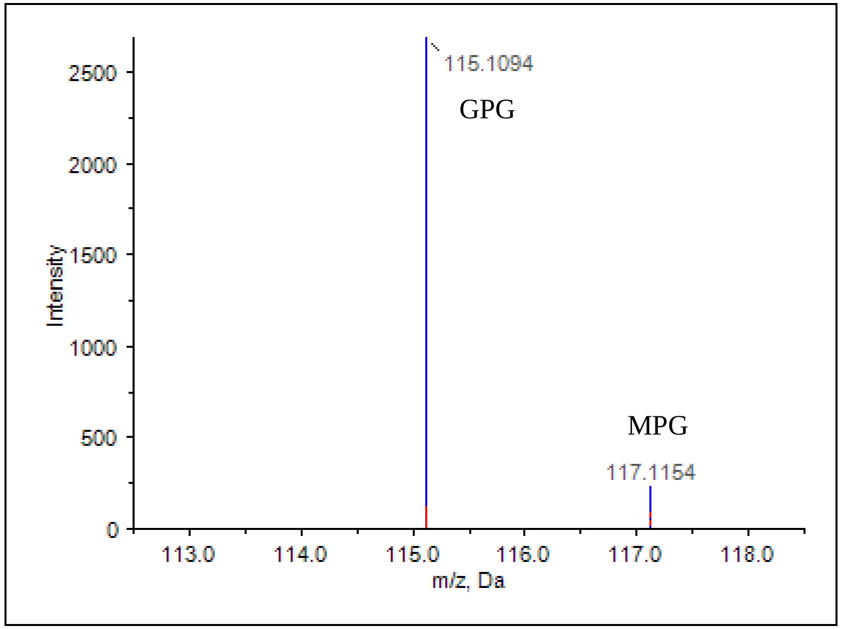

Precursor MS Region

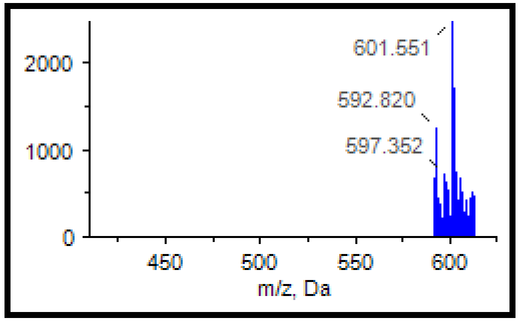

Accession: gi|115454901

Sequence:

QAFDDAIAELDTLGEESYK

Repeat 2: experiment 2

GPG (115) / MPG (117) = 10.00

Precursor mass (m/z): 601.55

IT4 QAFDDAIAELDTLGEESYK[IT4]

Residue	b	b+2	y	y+2
Q	273.1679	137.0876	2403.1806	1202.0939
A	344.2050	172.6062	2131.0199	1066.0136
F	491.2734	246.1404	2059.9828	1030.4950
D	606.3004	303.6538	1912.9144	956.9608
D	721.3273	361.1673	1797.8875	899.4474
A	792.3644	396.6859	1682.8605	841.9339
I	905.4485	453.2279	1611.8234	806.4153
A	976.4856	488.7464	1498.7393	749.8733
E	1105.5282	553.2677	1427.7022	714.3547
L	1218.6123	609.8098	1298.6596	649.8334
D	1333.6392	667.3232	1185.5756	593.2914
T	1434.6869	717.8471	1070.5486	535.7779
L	1547.7710	774.3891	969.5009	485.2541
G	1604.7924	802.8999	856.4169	428.7121
E	1733.8350	867.4211	799.3954	400.2013
E	1862.8776	931.9424	670.3528	335.6800
S	1949.9096	975.4585	541.3102	271.1588
Y	2112.9730	1056.9901	454.2782	227.6427
K[IT4]	2385.1700	1193.0886	291.2149	146.1111

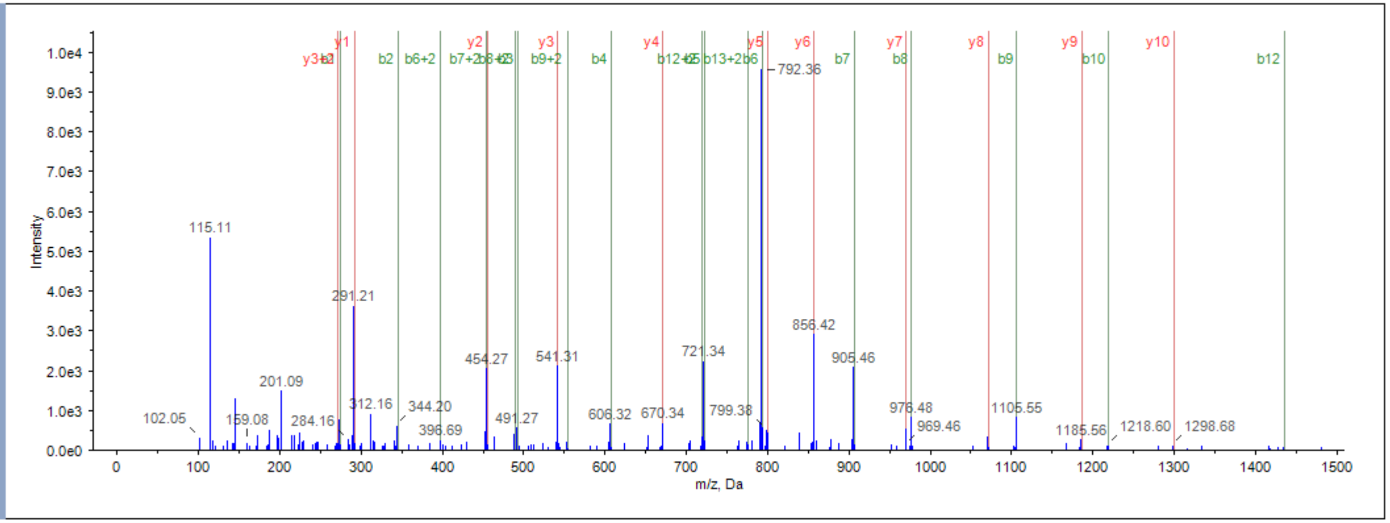

Accession: gi|115443865 **Sequence:** QIGAPYYIECSSK **Repeat 1:** experiment 1

GPG (117) / MPG (115) = 0.13 **Precursor mass (m/z):** 821.89

Peptide Quantitation Information

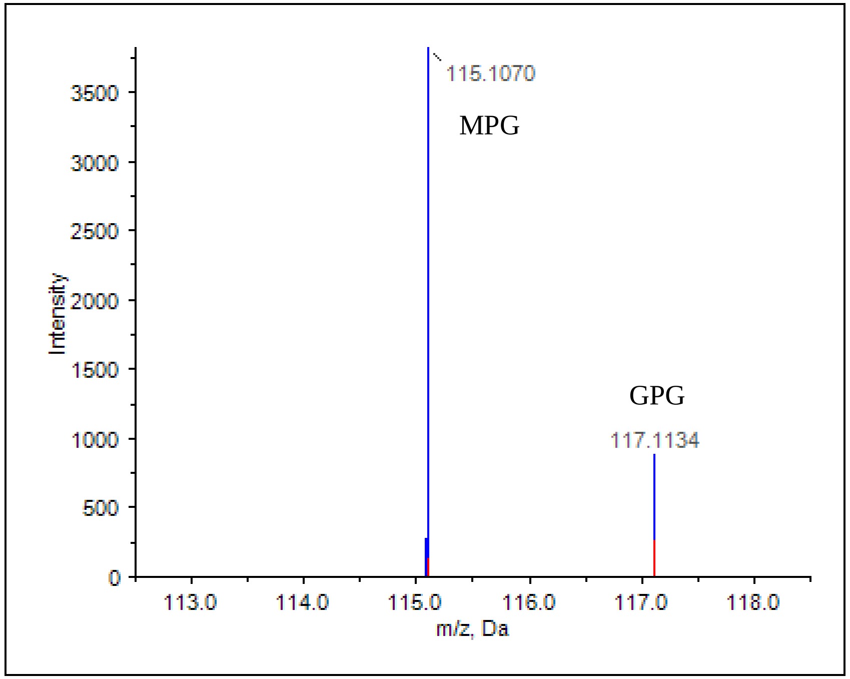

Precursor MS Region

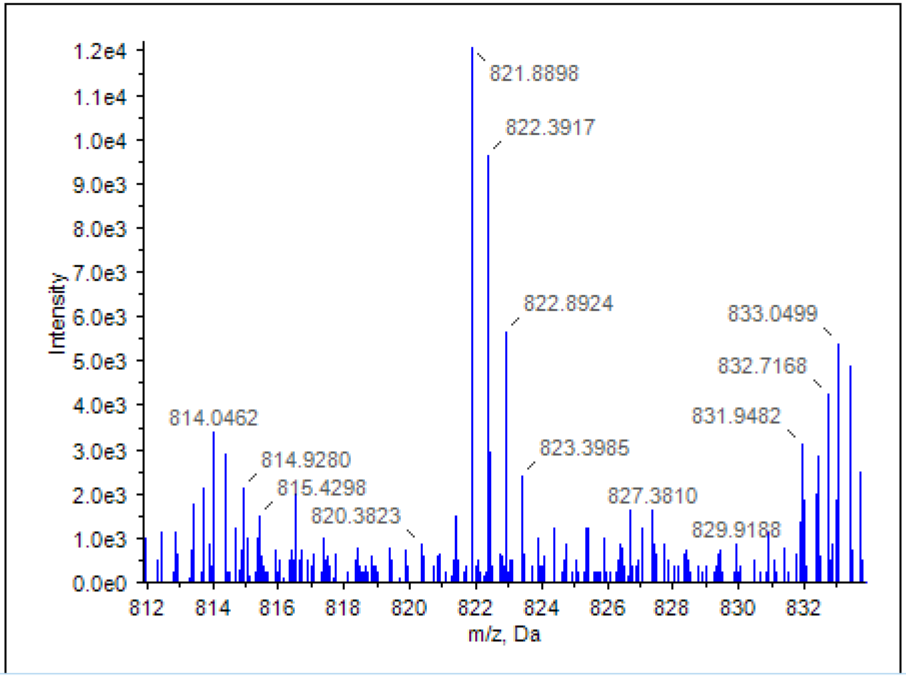

Fragmentation Evidence for Peptide

IT4 QIGAPYYIEC[CAM]SSK[IT4]

Residue	b	b+2	y	y+2
Q	273.1679	137.0876	1803.9189	902.4631
I	386.2520	193.6296	1531.7583	766.3828
G	443.2734	222.1404	1418.6742	709.8408
A	514.3106	257.6589	1361.6528	681.3300
P	611.3633	306.1853	1290.6156	645.8115
Y	774.4267	387.7170	1193.5629	597.2851
Y	937.4900	469.2486	1030.4996	515.7534
I	1050.5740	525.7907	867.4362	434.2218
E	1179.6166	590.3120	754.3522	377.6797
C[CAM]	1339.6473	670.3273	625.3096	313.1584
S	1426.6793	713.8433	465.2789	233.1431
S	1513.7113	757.3593	378.2469	189.6271
K[IT4]	1785.9084	893.4578	291.2149	146.1111

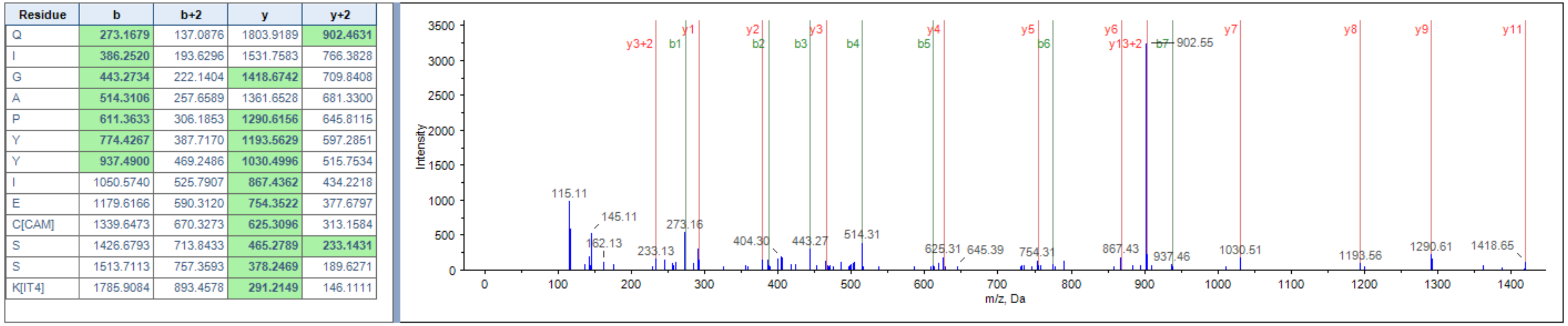

Peptide Quantitation Information

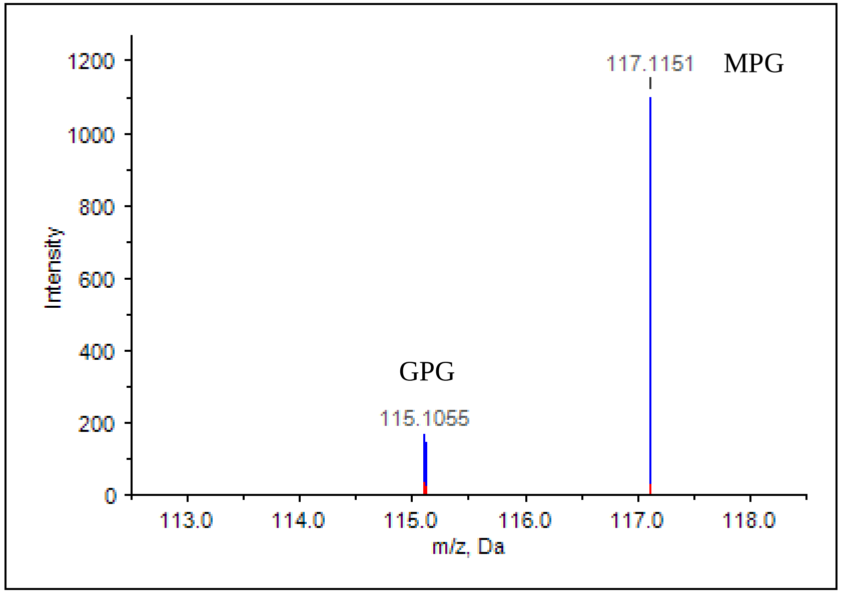

Precursor MS Region

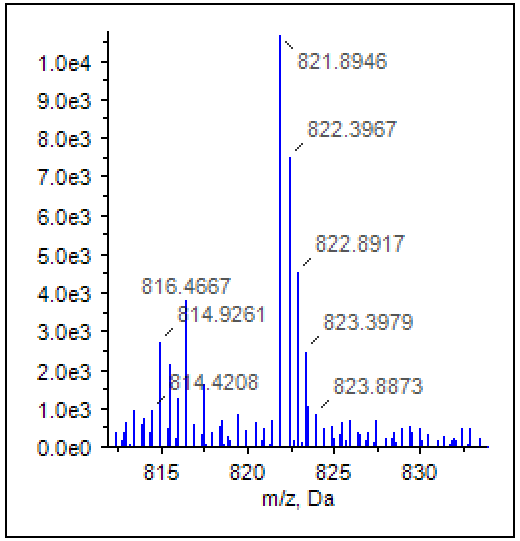

Accession: gi|115443865

Sequence:

QIGAPYYIECSSK

Repeat 2: experiment 2

GPG (115) / MPG (117) = 0.12

Precursor mass (m/z): 821.89

Fragmentation Evidence for Peptide

IT4 QIGAPYYIEC[CAM]SSK[IT4]

Residue	b	b+2	y	y+2
Q	273.1679	137.0876	1803.9189	902.4631
I	386.2520	193.6296	1531.7583	766.3828
G	443.2734	222.1404	1418.6742	709.8408
A	514.3106	257.6589	1361.6528	681.3300
P	611.3633	306.1853	1290.6156	645.8115
Y	774.4267	387.7170	1193.5629	597.2851
Y	937.4900	469.2486	1030.4996	515.7534
I	1050.5740	525.7907	867.4362	434.2218
E	1179.6166	590.3120	754.3522	377.6797
C[CAM]	1339.6473	670.3273	625.3096	313.1584
S	1426.6793	713.8433	465.2789	233.1431
S	1513.7113	757.3593	378.2469	189.6271
K[IT4]	1785.9084	893.4578	291.2149	146.1111

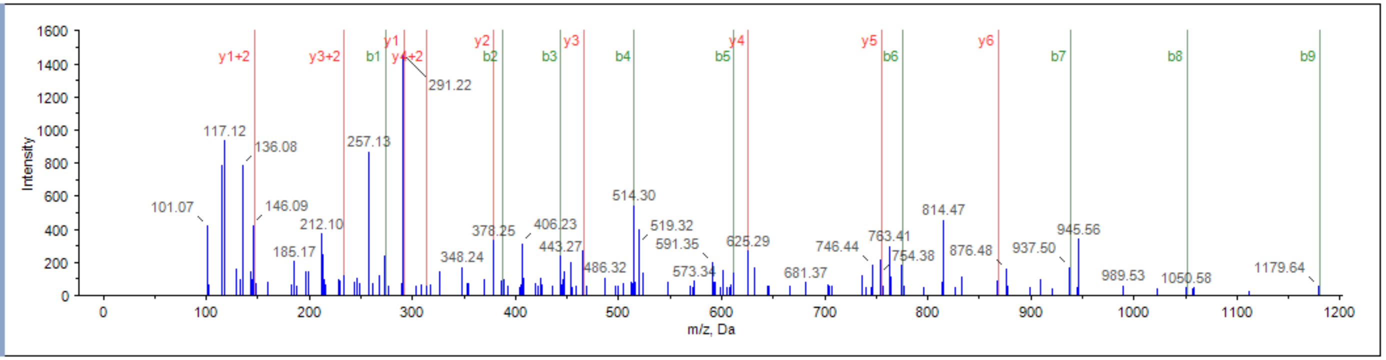

Supplement: Additional file 7: — Mass spectrometry spectra show the repeatability of iTRAQ quantitative information. (PDF 634 kb) [file 12870_2016_961_MOESM7_ESM.pdf]
