# Supplementary material for: Comparative proteomic analysis reveals a dynamic pollen plasma membrane protein map and the membrane landscape of receptor-like kinases and transporters important for pollen tube growth and interaction with pistils in rice
Source: BMC Plant Biol. 2017 Jan 5;17:2. doi: 10.1186/s12870-016-0961-7 (PMC5217431; doi:10.1186/s12870-016-0961-7)
Supplement: Additional file 10: — Western blot validation of the iTRAQ quantitative information. (PDF 94 kb) [file 12870_2016_961_MOESM10_ESM.pdf]

Additional file 10. Western blot validation of the iTRAQ quantitative information.

protein	iTRAQ [a] (GPG/MPG)				Western blot [b] (GPG/MPG)				
	**Exp 1**	**Exp 2**	**mean**	**S.D.**	**1**	**2**	**3**	**mean**	**S.D.**
eIF 4a	5.97	5.50	5.73	0.34	7.99	6.00	6.20	6.73	1.10
GAPDH	2.11	2.00	2.06	0.08	1.84	2.13	2.21	2.06	0.19
Band_7	0.94	0.94	0.94	0.00	0.83	0.89	0.93	0.88	0.05
Sar	0.26	0.23	0.25	0.02	0.32	0.28	0.30	0.30	0.02
Correlation coefficient between iTRAQ quantitation and WB quantitation = 0.9983									

a, Protein expression change ratio (GPG/MPG) quantified by iTRAQ.

b, Optical density ratio (GPG/MPG) of Western blot bands measured by Image-Pro Plus v6.0.

eIF 4a, the eukaryotic initiation factor-4a from *Oryza sativa*, gi|115444197; GAPDH, glyceraldehyde-3-phosphate dehydrogenase from *Oryza sativa*, gi|115459078; Band_7, flotillin like protein from *Oryza sativa*, gi|48716660; Sar, ras-related protein1 from *Arabidopsis thaliana*, AT3G62560, which shares 93% amino acid identity with rice gi|115436368.
