# Supplementary material for: Comparative proteomic analysis reveals a dynamic pollen plasma membrane protein map and the membrane landscape of receptor-like kinases and transporters important for pollen tube growth and interaction with pistils in rice
Source: BMC Plant Biol. 2017 Jan 5;17:2. doi: 10.1186/s12870-016-0961-7 (PMC5217431; doi:10.1186/s12870-016-0961-7)
Supplement: Additional file 15: — Multiple sequence alignment of CrRLK1Ls. Sequence alignments of identified rice pollen CrRLK1Ls gi|56783691, gi|115462979 and gi|25553554, and functionally known HERK1 (AT3G46290), HERK2 (AT1G30570), THE1 (AT5G54380), ANX1 (AT3G04690), ANX2 (AT5G28680) and FER (AT3G51550) from Arabidopsis. These CrRLK1Ls have a variable malectin domain (underlined in purple) and highly conserved kinase domain (in red) along with one transmembrane domain (in orange) predicted by SMART. Identical and similar amino acids are shaded in blue and yellow. (PDF 18045 kb) [file 12870_2016_961_MOESM15_ESM.pdf]

```
HERK1         ---MGIEKFETFILISTISILLCICHG-------------------FTPVDNYLINCGSPTNGT--LMGRIFLSDKLSSKLLT---SSKEILASVG--GNSG-SDIYHTARVFTEVSSYK 90
HERK2         ---MSKLRKKYLEHLLCVLIFFTYVIG-----------------YGEAQSKSFLVDCGSNATTE--VDGRTWVGDLSPNKSVTLQGFDAITASTSK--GSSVYAEIYKTARVFDAVLNYT 96
THE1          -----MVFTKSLLVLLWFLSCYTTTTS----------------SALFNPPDNYLISCGSSQNIT--FQNRIFVPDSLHSSLVLKIGNSSVATSTTS--NNST-NSIYQTARVFSSLASYR 94
ANX1          MSGKTR----ILF-FLTCLSFLLVFPT-----------------RSNG--QDLALSCGTSEASA-DQDKKWEPDTK---FLKTG--NSIHATATYQDPSLLSTVPYMTARIFTAPATYE 90
ANX2          MNEKLR----ILFSFLCFFYVLLVSPS-----------------QSNG--QDISLSCGASEPAV-DQDKKWEPDTK---FLKTP--NTVHAPATYQDPSLLSTVPYMTSRIFTAPATYE 91
FER           MKITEGRFRLSLLLLLLLISAATLISA-----------------ADYSPTEKILLNCGGGASNLTDTDNRIWISDVK-SKFLSSSSEDSKTSPALTQDPSVP-EVPYMTARVFRSPFTYT 101
gi|56783691   MAGRVRCSPAVALALVVAAAAAAAATA-----------------KLYSPADRILVNCGSTTDGL-DAEGRRWVADATNDTWLTDSGKSSIMAAADELETMLPSSIPYMTARVFTMDTVYN 102
gi|115462979  MASRR----HVLIAALIMVGVLEFANA-----------------DKYKPTESILVNCGSDKEGQ-DIDGRKWLSDKD-SKWLIDGEKSSIMANADFQDPSLPSPVPYMTARVFTKETMYN 97
gi|25553554   MRRRGRMIPPALLLAAAVAAALATAVSGQGRPVTESGAQTAPTPSTFTPKDNFLIDCGSTSPVT--TGGKVYKTDAQSNSLLS--AKDAIKVATTD--ADVP-SPLYLTARIFRDEAVYS 113

HERK1         FSVTR-GRHWVRLYFNPFDYQNFKMGSAKFAVSSQ-SHVLLSDFTVT---------------SSK---VVKEYSLN-VTTNDLVLTFTPS---SGSFAFVNAIEVISIPDTLITG--SPR 184
HERK2         FEGITQGNYFVRLHFSPFAIENHNVNESSFSVFAD-GLRLMLDINIAGEIAHKNLILESTGHNATASSLVKEFLLP-TGPGKLVLSFIPE---KGSFGFVNAIEIVSVDDKLFKE--SVT 209
THE1          FKITSLGRHWIRLHFSPINNSTWNLTSASITVVTE-DFVLLNNFSFN---------------NFNGSYIFKEYTVN-VTSEFLTLSFIPS---NNSVVFVNAIEVVSVPDNLIPD--QAL 192
ANX1          IPIKGDKRHLLRLYFYPSTYTGLNISNSYFTVEAN-DVTLLSNFSAA-----------ITCQALTQAYLVKEYSLAPTDKDVLSIKFTPSDKYRDAFAFINGIEVIQMPELFD----TAA 194
ANX2          IPVKGDKRHMLRLHFYPSTYTGLNILDSYFSVAAN-DLTLLSNFSAA-----------ITCQALTQAYLVREYSLAPSEKDVLSIIFTPSDKHPKAFAFINGIEVIPMPELFD----TAS 195
FER           FPVA-SGRKFVRLYFYPNSYDGLNATNSLFSVSFG-PYTLLKNFSAS-----------QTAEALTYAFIIKEFVVN-VEGGTLNMTFTPESAPSNAYAFVNGIEVTSMPDMYSSTDGTLT 207
gi|56783691   FTVNPRDRHWIRLHFYPSSYNGLEPQDFRFSVFTTTGYTLLHNFSVY-----------FTTKALTQAYLIREYSLPRVPEGHFGVTFSPSPMMNVTYAFVNGIEVISMPDMFNN---PAT 208
gi|115462979  FSVG-EERHWVRLHFYPASYHDLPAENFFFSVSTSTGITLLKNFSVY-----------ITAKALSQAYIIREFTLPPSTTGSLSLIFTPTAMNNASYAFVNGIEIISMPNIFSQ---AAA 202
gi|25553554   FPLTVPGWHFVRLYLFPLKNSDFDLTTATFTVSTD-TNVLLHSFTAE---------------NKP---VMKEFLVN-ATENHLAVKFYPL---KGSAAFINAIEVVNAPDELITD--MAM 208

HERK1         FVGNPAQFPDMSMQGLETIHRVNMGGPLVAS-NND-TLTRTWVPDSEFLLEKNLAKSMSKFSTVNFVPGYAT---EDSAPRTVYGSCTEMNSADNPNSIFNVTWEFDVDPGFQYYFRFHF 299
HERK2         KVGGSEVELGLGGRGIETMYRLNVGGPKLGP-SKDLKLYRTWETDLSYMVIENAGVEVKN-SSNITYALADD---SPVAPLLVYETARMMSNTEVLEKRFNISWKFEVDPNFDYLVRLHF 324
THE1          ALNPSTPFSGLSLLAFETVYRLNMGGPLLTS-QND-TLGRQWDNDAEYLHVNSSVLVVTANPSSIKYSPSVT---QETAPNMVYATADTMGDANVASPSFNVTWVLPVDPDFRYFVRVHF 307
ANX1          LVGFTDQTMDAKTANLQSMFRLNVGGQDIPGSQDSGGLTRTWYNDAPYIFSAGLGVTLQASNNFRINYQNMP---VSIAPADIYKTARSQGPNGDINLKSNLTWMFQIDKNFTYILRLHF 311
ANX2          LVGFSDQTSDTKTANLQTMFRLNVGGQDIPGSQDSGGLTRTWYNDAPYIFSAGLGVTLQASNNFRIDYQKMP---VSTAPADVYKTARSQGPNGDINMKSNLTWMFQVDTNFTYIMRLHF 312
FER           MVGSSGSVTIDNSTALENVYRLNVGGNDISPSADTG-LYRSWYDDQPYIFGAGLGIPETADPNMTIKYPTGT--PTYVAPVDVYSTARSMGPTAQINLNYNLTWIFSIDSGFTYLVRLHF 324
gi|56783691   MVGFADQTADVSAAAFQTMYRLNVGGAYIPPSNDSG-LTRPWYDDTPFVQGPLRGLVYNAGPHFHIKYPSD--AAEYAAPPEVYLGGRSMGRDQRLNQNSNLTWSLHVECNFTYVVRLHF 325
gi|115462979  SVDIAGNEVSTTDSSLQTIYRLNVGGSYVAPTNDSG-LSRDWYDDTPYIYGAAVGVTYQANDTVQIKYPKNDPDAEYAAPASVYLTSRSMGPDPKVNKNYKLTWVFEVDGNFTYIVRLHF 321
gi|25553554   GIAPVGEMTGLAEAAYQVVYRINVGGPAIAP-DKD-TLGRQWDVDAPYVQSKEAVKDVSVPVGNIKFPDGTS---KLVAPAQVYASCAKMADAGVGSPSFNMSWKMEVDPAFGYLVRLFF 323

HERK1         CDIVS--LSLNQLYFNLYVDSMVAATD---IDLSTLVDNTLAGAYSMDFVT--QTPKGS--NKVRVSIGPST-VHTDYPNAIVNGLEIMKMNNSKGQLSTGTFVPG-------------- 395
HERK2         CELLV--DKQNQRIFRIYINNQTAAGN---FDIFAHAG-GKNKGIYQDYLD--PVSSKN--DVLWIQLGPDS-SVGASGDALLSGLEIFKLSKN-GNLAHLIRFDSTGHS---------- 422
THE1          CDIVS--QALNTLVFNLYVNDDLALGS---LDLSTLTN-GLKVPYFKDFIS--NGSVESS-GVLTVSVGPD--SQADITNATMNGLEVLKISNEAKSLSGVSSVKSLLPG---------- 406
ANX1          CEFQ--LSKINQKVFNIYINNRTAQADTTPADIIGWTG-EKGIPMYKDYAIYVDANNGG--EEITLQMTPSTFGQPEYYDSSLNGLEIFKMD-TMKNLAGPNPEPSPMQAEEEV------ 419
ANX2          CEFQ--LAKINQKVFNIFINNRTAQGDTNPADILGWTG-GKGIPTYKDYAIYVDANTGGGGEEISLQMTPSTFGQPEYYDSQLNGLEIFKID-TMKNLAGPNPKPSPMQANEDV------ 422
FER           CEVSSNITKINQRVFTIYLNNQTAEPE---ADVIAWTS-SNGVPFHKDYVVNPPEGNGQ--QDLWLALHPNPVNKPEYYDSLLNGVEIFKMNTSDGNLAGTNPIPGPQVTADPSK---VL 435
gi|56783691   CELQ--LIHGNQRVFDIYINNRTAQTD---VDVLEMAT-ERGVPVYKDYAVRLSNDTAD--EHLWVAVHPSVMLRPQFYDAILNGLEVFKVNNTGGSLASPDPVPYKLLAEKELGWGGPP 437
gi|115462979  CELL--LSKPNQRVFDILINNKTAQSG---ADVIGWG--GQFVPVYKDYATIMPGGAGD--KVLWVQLMPNVGSGSEFFDSLLNGLEIFKMSDSSGNLAGPNPDPSKLLEEAESSAQG-- 430
gi|25553554   ADIVS--KSMNDLYFNVFVNGRKAISG---LDLSTVTG-ELSAAYYKDIVV--NSSIAT--DKLSIQVGPMG-EDTGRVDALLSGVEVLKMSNSVGSLDGEFGVDGKKADD--------- 423

HERK1         --SSSSSKSNLGLIVGSAIGSLLAVVFLGSCFVLYKKRKRGQDGHSKT-------WMPFSING----TSMGSKYSNGTTLTS---ITTNANYRIPFAAVKDATNNFDESRNIGVGGFGKV 499
HERK2         --VSDSKMRIIWISVGAGIAIIIFFVFLGILVVCLCKKRRSKSDESKN---NPPGWRPLFLHVN---NSTANAKATGGSLRLNTLAASTMGRKFTLAEIRAATKNFDDGLAIGVGGFGKV 534
THE1          --GSGSKSKKKAVIIGSLVGAVTLILLIAVCCYCCLVASRKQRSTSPQEGGNGHPWLPLPLYGLSQTLTKSTASHKSATASCISLASTHLGRCFMFQEIMDATNKFDESSLLGVGGFGRV 524
ANX1          KKEFKNEKR-HAFIIGSAGGVLAV-LIGALCFT-AYKKKQ----GYQGGDSHTSSWLPIYGNSTTSGTKST-ISGKSNNGSHLSNLAAGLCRRFSLPEIKHGTQNFDDSNVIGVGGFGKV 531
ANX2          KKDFQGDKRITAFVIGSAGGVAAV-LFCALCFT-MYQRKR----KFSGSDSHTSSWLPIYGNSHTSATKST-ISGKSNNGSHLSNLAAGLCRRFSLSEIKHGTHNFDESNVIGVGGFGKV 535
FER           RPTTRKSKSNTAIIAGAASGAVVLALIIGFCVFGAYRRRKRG--DYQPASDATSGWLPLSLYGNSHSAGSA-KTNTTGS--YASSLPSNLCRHFSFAEIKAATKNFDESRVLGVGGFGKV 550
gi|56783691   EFSTDNPANMASVMGGTAGGAAAAGIVAAICVV-VYSNKRSKKLGGGGADSHTSAWLPLY--HSHTS-------GKSSG--HITANIAGMCRHFSFAEIKAATKNFSNDLAIGVGGFGVV 545
gi|115462979  KFKSKPSNLKATVIGGAAGGAAAFGIVAAICIV-VYQSKKRKVLNN--SASHSSGWLPVYGGNSHTSTSKS-SGGRSAA--LINPNITAMCRHFSFGEIKSATKNFDESLVIGVGGFGKV 544
gi|25553554   --GSGSRKAVAAVGFAMMFGAFAGLGAMAVKWYKRPQDWERRNSFSS--------WLLPIHTGQSFTTSKGGSSKSGYTFS----STLGLGRFFSFAEIQAATKNFEESAIIGVGGFGNV 529

HERK1         YKGELND-GTKVAVKRGNPKSQQGLAEFRTEIEMLSQFRHRHLVSLIGYCDENNEMILIYEYMENGTVKSHLYG-SGLPSLTWKQRLEICIGAARGLHYLHTGDSKPVIHRDVKSANILL 617
HERK2         YRGELED-GTLIAIKRATPHSQQGLAEFETEIVMLSRLRHRHLVSLIGFCDEHNEMILVYEYMANGTLRSHLFG-SNLPPLSWKQRLEACIGSARGLHYLHTGSERGIIHRDVKTTNILL 652
THE1          YKGTLED-GTKVAVKRGNPRSEQGMAEFRTEIEMLSKLRHRHLVSLIGYCDERSEMILVYEYMANGPLRSHLYG-ADLPPLSWKQRLEICIGAARGLHYLHTGASQSIIHRDVKTTNILL 642
ANX1          YKGVIDG-TTKVAVKKSNPNSEQGLNEFETEIELLSRLRHKHLVSLIGYCDEGGEMCLVYDYMAFGTLREHLYN-TKKPQLTWKRRLEIAIGAARGLHYLHTGAKYTIIHRDVKTTNILV 649
ANX2          YKGVIDG-GTKVAIKKSNPNSEQGLNEFETEIELLSRLRHKHLVSLIGYCDEGGEMCLIYDYMSLGTLREHLYN-TKRPQLTWKRRLEIAIGAARGLHYLHTGAKYTIIHRDVKTTNILL 653
FER           YRGEIDGGTTKVAIKRGNPMSEQGVHEFQTEIEMLSKLRHRHLVSLIGYCEENCEMILVYDYMAHGTMREHLYK-TQNPSLPWKQRLEICIGAARGLHYLHTGAKHTIIHRDVKTTNILL 669
gi|56783691   YRGVVDG-DVKVAVKRSNPSSEQGITEFQTEVEMLSKLRHRHLVSLIGFCEEDGEMVLVYDYMEHGTLREHLYHNGGKPTLSWRHRLDICIGAARGLHYLHTGAKYTIIHRDVKTTNILV 664
gi|115462979  YRGVVDG-DTKVAIKRSNPSSEQGVLEFQTEIEMLSKLRHKHLVSLIGCCEDEGEMILVYDYMAHGTLREHLYK-GGKPALSWKQRLEITIGAARGLHYLHTGAKYTIIHRDVKTTNILV 662
gi|25553554   YIGEIDD-GTKVAVKRGNPQSEQGINEFNTEIQMLSKLRHRHLVSLIGYCDENAEMILVYEYMHNGPFRDHIYG-KDLPALTWKQRLEICIGAARGLHYLHTGTAQGIIHRDVKTTNILL 647

HERK1         DENFMAKVADFGLSKTGPE-LDQTHVSTAVKGSFGYLDPEYFRRQQLTDKSDVYSFGVVLFEVLCARPVIDPTLPREMVNLAEWAMKWQKKGQLDQIIDQSLRGNIRPDSLRKFAETGEK 736
HERK2         DENFVAKMSDFGLSKAGPS-MDHTHVSTAVKGSFGYLDPEYFRRQQLTEKSDVYSFGVVLFEAVCARAVINPTLPKDQINLAEWALSWQKQRNLESIIDSNLRGNYSPESLEKYGEIAEK 771
THE1          DENLVAKVADFGLSKTGPS-LDQTHVSTAVKGSFGYLDPEYFRRQQLTEKSDVYSFGVVLMEVLCCRPALNPVLPREQVNIAEWAMAWQKKGLLDQIMDSNLTGKVNPASLKKFGETAEK 761
ANX1          DENWVAKVSDFGLSKTGPN-MNGGHVTTVVKGSFGYLDPEYFRRQQLTEKSDVYSFGVVLFEILCARPALNPSLPKEQVSLGDWAMNCKRKGNLEDIIDPNLKGKINAECLKKFADTAEK 768
ANX2          DENWVAKVSDFGLSKTGPN-MNGGHVTTVVKGSFGYLDPEYFRRQQLTEKSDVYSFGVVLFEVLCARPALNPSLSKEQVSLGDWAMNCKRKGTLEDIIDPNLKGKINPECLKKFADTAEK 772
FER           DEKWVAKVSDFGLSKTGPT-LDHTHVSTVVKGSFGYLDPEYFRRQQLTEKSDVYSFGVVLFEALCARPALNPTLAKEQVSLAEWAPYCYKKGMLDQIVDPYLKGKITPECFKKFAETAMK 788
gi|56783691   DDNWVAKVSDFGLSKSGPTTLNQSHVSTVVKGSFGYLDPEYYRRQQLTDKSDVYSFGVVLFEVLMARPALDPALPRDQVSLADYALACKRGGALPDVVDPAIRDQIAPECLAKFADTAEK 784
gi|115462979  DEKWVAKVSDFGLSKTGPTAMNQTHVSTMVKGSFGYLDPEYFRRQQLTEKSDVYSFGVVLFEVLCARPALNPSLPREQVSLADHAMSCQRKGTLHDIIDPLLNGKIAPDCLKKFAETAEK 782
gi|25553554   DDNFVAKVSDFGLSKDGPG-MNQLHVSTAVKGSFGYLDPEYFRCQQLTDKSDVYSFGVVLLETLCARPPIDPQLPREQVSLAEWGMQWKRKGLIEKIMDPKLAGTVNQESLNKFAEAAEK 766

HERK1         CLADYGVDRPSMGDVLWNLEYALQLQEAVID-G--------------EPEDNSTNMIGELPPQINNFSQGDTSVNVPGTAG-RFEESSIDDLSGVSMSKVFSQLVKSEGR 830
HERK2         CLADEGKNRPMMGEVLWSLEYVLQIHEAWLR-K--------------QNGENSFSSSQ-------AVEEAPESFT--------LPACSNQDSSETEQSQTGSALHNSA-- 849
THE1          CLAEYGVDRPSMGDVLWNLEYALQLEETSSALM--------------EPDDNSTNHIPGIP--MAPMEPFDNSMSIIDRGGVNSGTGTDDDAEDATTSAVFSQLVHPRGR 855
ANX1          CLNDSGLERPTMGDVLWNLEFALQLQETADGTRHRTP----------NNGGSSEDLGRGGMAVNV---AGRDDVS----------DLSSED-----NTEIFSQIVNPKGR 850
ANX2          CLSDSGLDRPTMGDVLWNLEFALQLQETADGSRHRTP----------SNGGGSVDLGGGGGGVTVNISAGESDLGD---------DLSSEE-----NSGIFSQIVNPKGR 858
FER           CVLDQGIERPSMGDVLWNLEFALQLQESAEENGKGVCGDMDMDEIKYDDGNCKGKNDKSSDVYEGNVTDSRSSGIDMSIGG---RSLASEDSDGLTPSAVFSQIMNPKGR 895
gi|56783691   CLSENGTERPTMGDVLWNLESAMHFQDAFDAAAGRPVPAL------DAAAGSSSHLDDGSTASINTLATSSTSHPHEPCVD---VVLEPDDV--VAERATFSQLVQPTGR 883
gi|115462979  CLADHGVDRPSMGDVLWNLEFALQMQETFENGG--------------KTEGADSTSDSTTTSVADSMAANAAALS---LIS---EDMDEED---IANSVVFSQLVRPTGR 869
gi|25553554   CLAEFGSDRISMGDVLWNLEYALQLQDANPP----------------EGADKPADHDG------AGAAPATSSG---------SGVSTVPDVSTTAAGEMFAQLADMKGR 845
```
